# TMEM161B regulates cerebral cortical gyration, Sonic Hedgehog signaling, and ciliary structure in the developing central nervous system

**DOI:** 10.1073/pnas.2209964120

**Published:** 2023-01-20

**Authors:** Shyam K. Akula, Jack H. Marciano, Youngshin Lim, David Exposito-Alonso, Norma K. Hylton, Grace H. Hwang, Jennifer E. Neil, Nicole Dominado, Rosie K. Bunton-Stasyshyn, Janet H. T. Song, Maya Talukdar, Aloisia Schmid, Lydia Teboul, Alisa Mo, Taehwan Shin, Benjamin Finander, Samantha G. Beck, Rebecca C. Yeh, Aoi Otani, Xuyu Qian, Ellen M. DeGennaro, Fowzan S. Alkuraya, Sateesh Maddirevula, Gregory D. Cascino, Caterina Giannini, Undiagnosed Diseases Network, Lindsay C. Burrage, Jill A. Rosenfield, Shamika Ketkar, Gary D. Clark, Carlos Bacino, Richard A. Lewis, Rosalind A. Segal, J. Fernando Bazan, Kelly A. Smith, Jeffrey A. Golden, Ginam Cho, Christopher A. Walsh

**Affiliations:** ^a^ Division of Genetics and Genomics, Manton Center for Orphan Disease Research, Boston Children’s Hospital, Boston, MA 02115; ^b^ Harvard-Massachusetts Institute of Technology MD/PhD Program, Program in Neuroscience, Harvard Medical School, Boston, MA 02115; ^c^ Howard Hughes Medical Institute, Boston Children’s Hospital Boston, Boston, MA 02115; ^d^ Department of Pediatrics, Harvard Medical School, Boston, MA 02115; ^e^ Department of Neurology, Harvard Medical School, Boston, MA 02115; ^f^ Department of Pathology and Laboratory Medicine, Cedars-Sinai Medical Center, Los Angeles, CA 90048; ^g^ Department of Cancer Biology, Dana-Farber Cancer Institute, Boston, MA 02115; ^h^ Department of Neurobiology, Harvard Medical School, Boston, MA 02115; ^i^ Department of Anatomy & Physiology, The University of Melbourne, Melbourne, VIC 3010, Australia; ^j^ Mary Lyon Centre, United Kingdom Medical Research Council Harwell, Didcot, Oxfordshire, OX11 0RD, UK; ^k^ Department of Physics/Electron Microscopy Core, Northeastern University, Boston, MA 02115; ^l^ Department of Translational Genomics, Center for Genomic Medicine, King Faisal Specialist Hospital and Research Center, 11564 Riyadh, Saudi Arabia; ^m^ Department of Neurology, Mayo Clinic, Rochester, MN 55905; ^n^ Department of Laboratory Medicine and Pathology, Mayo Clinic, Rochester, MN 55905; ^o^ Department of Molecular and Human Genetics, Baylor College of Medicine, Houston, TX 77030; ^p^ Departments of Pediatrics, Baylor College of Medicine, Houston, TX 77030; ^q^ Neurology, Baylor College of Medicine, Houston, TX 77030; ^r^ Neuroscience, Baylor College of Medicine, Houston, TX 77030; ^s^ Unit for Structural Biology, Vlaams Instituut voor Biotechnologie-UGent Center for Inflammation Research, 9052 Ghent, Belgium

**Keywords:** Sonic Hedgehog, polymicrogyria, holoprosencephaly, primary cilia, cortical gyration

## Abstract

Sonic hedgehog signaling regulates processes of embryonic development across multiple tissues, yet factors regulating context-specific Shh signaling remain poorly understood. Exome sequencing of families with polymicrogyria (disordered cortical folding) revealed multiple individuals with biallelic deleterious variants in *TMEM161B*, which encodes a multi-pass transmembrane protein of unknown function. *Tmem161b* null mice demonstrated holoprosencephaly, craniofacial midline defects, eye defects, and spinal cord patterning changes consistent with impaired Shh signaling, but were without limb defects, suggesting a CNS-specific role of Tmem161b. *Tmem161b* depletion impaired the response to Smoothened activation in vitro and disrupted cortical histogenesis in vivo in both mouse and ferret models, including leading to abnormal gyration in the ferret model. Tmem161b localizes non-exclusively to the primary cilium, and scanning electron microscopy revealed shortened, dysmorphic, and ballooned ventricular zone cilia in the *Tmem161b* null mouse, suggesting that the Shh-related phenotypes may reflect ciliary dysfunction. Our data identify *TMEM161B* as a regulator of cerebral cortical gyration, as involved in primary ciliary structure, as a regulator of Shh signaling, and further implicate Shh signaling in human gyral development.

## Significance

By evaluating children with cortical folding malformations, we identified *TMEM161B*, a gene with previously unknown function that is part of no known protein superfamily. In this work, we show that disrupting *Tmem161b* in utero is sufficient to lead to gyration abnormalities in a ferret model. We also characterized a *Tmem161b* null mouse that demonstrated developmental abnormalities including sequelae of abnormal Sonic Hedgehog signaling as well as alterations of primary ciliary structure. These connections help provide hypotheses for the cellular functions of *Tmem161b*, but also implicate Sonic Hedgehog signaling in promoting the normal folding of the human cortex.

Sonic Hedgehog (Shh), among many other roles in the developing central nervous system (CNS), patterns the dorsal–ventral axis of the spinal cord and brain (1–3) and is involved in patterning of the ocular primordium (4). Consequently, pathogenic variants in genes encoding Shh signaling components such as Smoothened (loss of function/haploinsufficiency) or Patched (gain of function) are associated with catastrophic midline defect phenotypes including holoprosencephaly (HPE), craniofacial defects (3, 5), and disorders of eye development (4, 6). Shh signaling has now been studied for more than 30 years, and interactions of the core pathway components like the receptor Smoothened or Patched, have been described at an increasingly granular level (7). In contrast, how Shh signaling is regulated within specific tissue types or locations, such as in the CNS versus the limb, remains incompletely understood.

While Shh has established roles in the early patterning of the CNS, emerging data also hint toward roles in the later processes of corticogenesis, specifically in the development of cortical gyration. Studies in ferret models have demonstrated that Shh signaling can influence local gyrification by affecting the proliferation of a specific outer radial glial (oRG) cell class, HOPX+ oRGs, enriched in gyrencephalic animals compared to mice (8–10). These experiments extend pioneer observations that ectopic constitutive Shh stimulation in developing mouse cortex is sufficient to form cortical folds (not true gyri) in the otherwise lissencephalic brain (11). However, how Shh signaling influences oRG dynamics and the mechanisms regulating its effect on the development of cortical gyration remain to be described.

Vertebrate Shh signaling depends on the primary cilium (12, 13), a hair-like microtubule-based organelle that serves as a compartmentalized signaling apparatus in eukaryotes. Shh signaling is vulnerable to disruptions in ciliary trafficking, compartmentalization, and ciliary exocytosis; disruptions in these processes lead to phenotypes similar to those resulting from pathogenic variants in genes encoding core Shh pathway components (3, 14, 15). Thus, exploring factors that, when disrupted, cause stereotypical phenotypes of defective Shh signaling, or that impact the function of the primary cilium, provides avenues to better understand Shh signaling mechanisms in multiple contexts.

Here, we identify individuals with variants in *TMEM161B* with diffuse polymicrogyria (PMG)—a disorder of cortical gyration—but with limited extra-CNS manifestations of disease. At the outset of our work, no biological functions had been ascribed to TMEM161B, although it was recently identified as a regulator of cardiac rhythm by tempering specific currents (16). *TMEM161B* belongs to a superfamily of proteins conserved down to single-celled eukaryotes, with unknown biochemical functions. We characterized a *Tmem161b* null mouse model and discovered multiple phenotypes suggestive of defective Shh signaling. We demonstrated that disruption of *Tmem161b* impairs normal response to Smoothened activation in vitro. In vivo knockdown of *Tmem161b* in the developing mouse and ferret brain by in utero electroporation caused disrupted neuronal positioning as well as abnormal local gyral formation in the ferret. Finally, we propose a cellular role for *TMEM161B* by finding dramatic structural disruptions of *Tmem161b* null primary cilia. Our data indicate that *TMEM161B* is essential for normal cortical gyral malformation and regulates Shh signaling in the developing mammalian CNS.

## Results

### Variants in TMEM161B Are Associated with Diffuse Polymicrogyria.

**Fig. 1.**
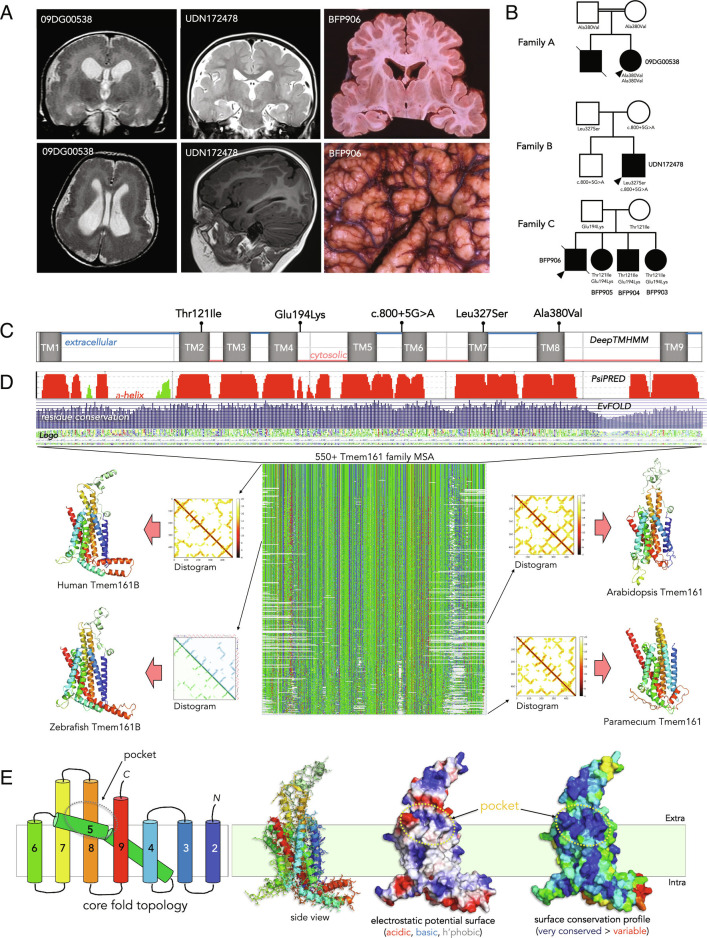
Missense variants in *TMEM161B*, a highly conserved 9-transmembrane domain protein, are associated with cortical malformation. (*A*) Brain MRIs and pathology of affected individuals display diffuse polymicrogyria. MRI images of 09DG00538 are at 2 y of age, MRI images of UDN172478 are at 3 y of age, and images of BFP906 are from postmortem examination at age 32 y. All three show a similar coronal brain contour and diffuse PMG across the entire cortex bilaterally. (*B*) Pedigrees of affected families. *TMEM161B* (NM_153354.4) genotypes are labeled where they were available and confirmed, black shading indicates affected or presumed affected individuals, tissue for BFP906 was unavailable for sequencing. The recessive inheritance and segregation of *TMEM161B* variants with disease supports a loss-of-function disease model. (*C*) Distribution of identified variants in *TMEM161B*. The missense variants identified are distributed throughout TMEM161B in highly conserved residues ( *SI Appendix*, Fig. S1*C* ), marked atop the TM helix prediction by DeepTMHMM (https://dtu.biolib.com/DeepTMHMM). (*D*) Multisequence alignment of *TMEM161B* orthologs across domains of eukaryotic life reveals deep conservation of orthologs across its 9TM chain architecture, suggesting a fundamental biological role. Structural models build with TrRosetta and Tfold (with attendant predicted distance matrices or distograms that highly resemble each other)––that agree well with more recent AlphaFold2 predictions––are drawn for the human TMEM161B as well as its zebrafish ortholog. More distant plant and amoeba TMEM161s likewise share the 9TM helix fold. (*E*) Modeling of the predicted structure of TMEM161B integrating evolutionary conservation data. TM1 could serve as an atypical signal peptide, for a net 8-TM domains in the mature protein. TMEM161s have eight predicted TM helices with an unusual, highly conserved, non-symmetric core fold topology that defines the TMEM161 superfamily. The most conserved feature is a shallow extracellular pocket bordered by TM helix extensions that just above the membrane, as labeled on the figure in both an electrostatic surface profile, and surface residue conservation profile of human TMEM161B.

As part of a larger study using exome sequencing (ES) to study genetic causes of polymicrogyria (PMG), we discovered multiple families with segregating biallelic variants in *TMEM161B*. Individual 09DG00538 (Family A, Fig. 1 *A*–*C* and *SI Appendix*, Fig. S1*C* ) presented at birth after normal delivery with intractable seizures, severe microcephaly, and hypotonia/spastic paresis; an MRI revealed diffuse PMG. ES uncovered a homozygous missense variant in *TMEM161B* (NM_153354.4)*,* c.1139C>T; p.(Ala380Val), at a highly conserved amino acid and predicted deleterious by three in silico tools [CADD (23.0), Mutation Taster (64, v2021), SIFT (0.02, v6.2.0)] ( *SI Appendix*, Fig. S1 *A* and *C* ). ES of Individual UDN172478 (Family B, Fig. 1 *A*–*C*), who has diffuse PMG, pachygyria and a similar brain shape to Individual 09DG00538, revealed compound heterozygous variants in *TMEM161B*: a missense variant, c.980T>C; p.(Leu327Ser), at a highly conserved amino acid and predicted deleterious by four in silico tools [CADD (28.4), Mutation Taster (58, v2021), SIFT (0.01, v6.2.0), PolyPhen-2 (0.999)]; and a splice region variant, c.800+5G>A [chr5(GRCh37):g.87501626C>T] having a CADD score of 24.6 and predicted by three in silico tools to abolish the upstream natural splice donor site of intron 8 [MaxEntScan (−100.0%), NNSPLICE (−98.0%), SpliceSiteFinder-like (−16.7%)] (Fig. 1 *A*–*C* and *SI Appendix*, Fig. S1*C* ). We conducted RNAseq in patient-derived fibroblasts and showed that this splice region variant leads to exon skipping ( *SI Appendix*, Fig. S1*D* ). Although the skipping of exon 8 is predicted to produce an in-frame transcript, it deletes the most highly conserved portion of the protein (see below). Finally, we identified compound heterozygous *TMEM161B* missense variants in individuals BFP903, BFP904, and BFP905 of a family of four similarly affected siblings. These missense variants are: c.362C>T; p.(Thr121Ile), affecting a moderately conserved amino acid and predicted deleterious by four in silico tools [CADD (24.6), Mutation Taster (73, v2021), SIFT (0.01, v6.2.0), PolyPhen-2 (0.999)]; and c.580G>A; p.(Glu194Lys), affecting a highly conserved amino acid and predicted deleterious by three in silico tools [CADD (24.8), Mutation Taster (52, v2021), SIFT (0.03, v6.2.0)] (Family C, Fig. 1 *A*–*C* and *SI Appendix*, Fig. S1*C* ). All four siblings presented with a similar neurological syndrome consisting of spastic hemiparesis, dysarthria, intellectual disability, and seizures. Diffuse PMG was discovered in the eldest sibling on autopsy after sudden cardiac death (Family C, Individual BFP906, Fig. 1 *A*–*C* and *SI Appendix*, Fig. S1*B* ). Brain imaging was unavailable on the other affected siblings. Across all families, the affected individuals for whom detailed phenotypic data were available have a striking coherence of diffuse PMG and brain shape. Further clinical details are provided in the supplement. The recessive inheritance, the presence of putative damaging alleles in heterozygosity only in the gnomAD database (17), the distribution of the four missense alleles spread across the protein, and the splice region variant that deletes the most highly conserved portion of the protein together imply that *TMEM161B*-related disease occurs via a loss-of-function mechanism. The similarity in presentation across multiple independent families establishes *TMEM161B* as associated with human cortical malformation, specifically PMG.

### TMEM161B Is Highly Conserved across Eukaryotes.

*TMEM161B* encodes a member of a previously uncharacterized protein superfamily thought to consist only of itself and its paralog *TMEM161A.* This superfamily has no sequence similarity to known classes of transmembrane (TM) proteins (e.g., receptors, channels, transporters, enzymes, etc.) nor annotated functional domains that might suggest molecular function. Most organisms examined have only a single ortholog of an ancestral TMEM161 protein; *TMEM161A* and *TMEM161B* arose from a duplication event of this ancestral gene that occurred between the invertebrate and vertebrate lineages (18). We have employed a series of increasingly accurate, deep-learning-assisted fold prediction algorithms, from TrRosetta (19) to AlphaFold2 (20), to model the three-dimensional structures of TMEM161 family members that were harvested from sequence databases by sensitive HHpred (21) searches that reveal homologs in plants and single-celled amoeba. These uncovered a deeply conserved common architectural plan of 9 TM helices with no obvious internal symmetry or central pore––though we can identify a preserved binding pocket nestled by helices on the extracellular face of all TMEM161s (Fig. 1 *D* and *E*). N-terminal TM1 showed a weak or equivocal signal peptide nature by SignalP5 (22). The protein substitution variants seen in the individuals presented all occur in highly conserved regions and disrupt core packing of TM helices (p.Thr121Ile, p.Leu327Ser) or cause charge reversal in another highly conserved pocket (p.Glu194Lys) ( *SI Appendix*, Fig. S1*A* ). The splicing variant is predicted to skip an exon that codes for TM5, the loss of which would likely disrupt the core fold and function of the protein. Therefore, the predicted effects on protein structure, along with pathogenicity predictions and patterns of inheritance identified in the families described suggest that all four missense alleles cause partial or complete loss of function.

### TMEM161B Is Expressed in the Developing Central Nervous System.

**Fig. 2.**
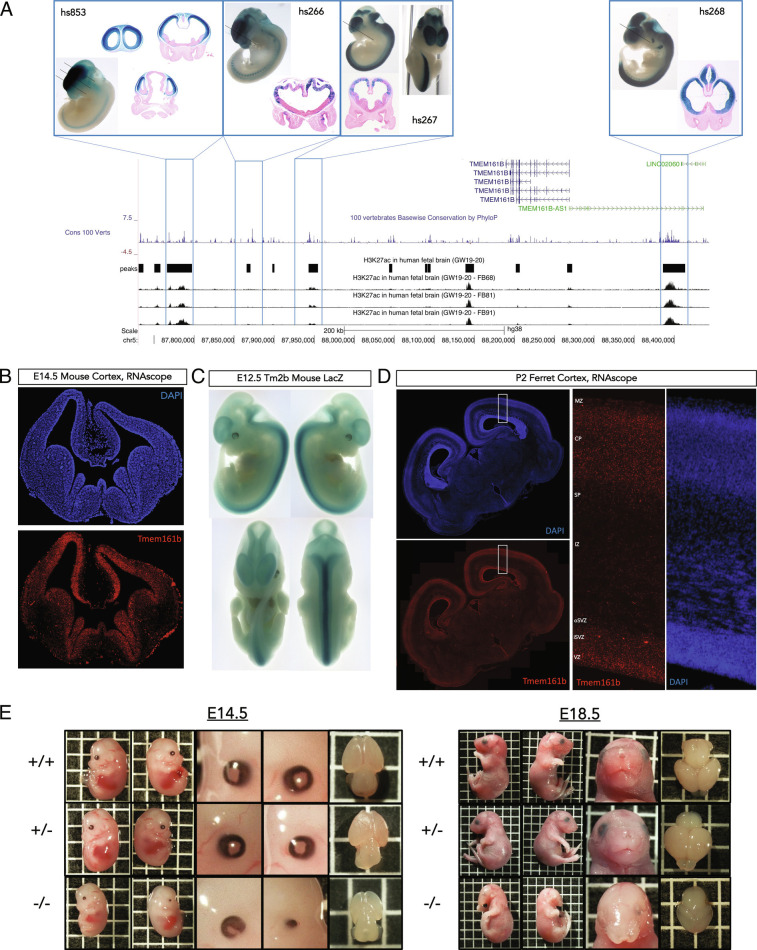
*TMEM161B* shows spatiotemporal expression enrichment for the developing CNS, and *Tmem161b* KO mouse embryos show gross abnormalities. (*A*) Multiple conserved active/open enhancers in developing CNS flank *TMEM161B*. Alignment of human fetal H3K27ac data with *TMEM161B* locus, with overlays of representative LacZ stains from the VISTA Enhancer Browser (24) demonstrating that several local H3K27ac peaks correspond to highly conserved enhancers expressed in the developing CNS. (*B*) RNAscope for *Tmem161b* in developing mouse brain. In situ hybridization using RNAscope against *Tmem161b* at E14.5 shows diffuse *Tmem161b* expression in developing brain with a bias towards ventricular zones in both ventral and dorsal forebrain, this expression pattern is concordant with the enhancer LacZ staining in Fig. 2*A*. (*C*) LacZ staining in *Tmem161b* ^tm2b/+^ embryos reveals enriched *Tmem161b* expression in the developing CNS. E12.5 embryos reveal high concentration of *Tmem161b*-driven LacZ expression in the developing CNS on top of diffuse low-level expression. (*D*) RNAscope for *Tmem161b* in P2 ferret brain. During neurogenesis and migration in the gyrencephalic ferret, *Tmem161b* is expressed and enriched slightly in the ventricular zone, but also shows expression across cortical plate. (*E*) *Tmem161b* null embryos display numerous anatomical abnormalities: KO mice were smaller, had a range of eye defects from coloboma to anophthalmia, and craniofacial defects. KO brains were smaller and demonstrated variable holoprosencephaly (more examples of all phenotypes in the supplement). Heterozygous mice were indistinguishable from WT mice. One box = 0.5 cm side length.

The spatial and temporal expression of *TMEM161B* is consistent with essential roles in CNS development. Analysis of chromatin accessibility in human fetal brain at GW19-20 (23) near *TMEM161B* showed multiple H3K27ac peaks near the transcription start site (TSS) of *TMEM161B* (Fig. 2*A*) and the divergent lncRNA that shares the same TSS, *TMEM161B-AS1*, implying CNS-specific enhancer regulation that affects these transcripts. Several of these peaks correspond to conserved enhancer sites that are expressed specifically in the developing CNS, validated by the VISTA enhancer project (24), which chooses enhancers to study due to their high degree of conservation (Fig. 2*A*). We found that the TSS of *TMEM161B* is in physical contact with these enhancers in both adult and fetal cortical neuronal cell types by analyzing proximity ligation-assisted chromatin immune precipitation sequencing (PLAC-seq) data (25–27) ( *SI Appendix*, Fig. S2*A* ). Bulk RNA seq of human frontal cortex shows higher *TMEM161B* expression in fetal tissue compared to adult tissue ( *SI Appendix*, Fig. S2*B* ) and scRNAseq in human fetal brain at GW17 (28) showed that, while *TMEM161B* is expressed at low levels in most cell types of the developing CNS, its expression is enriched in neural progenitor cell types relative to mature neurons ( *SI Appendix*, Fig. S3), and in HOPX+ oRGs compared to other radial glial populations ( *SI Appendix*, Fig. S3).

In situ hybridization against *Tmem161b* transcript at embryonic day 14.5 (E14.5) in mice showed a diffuse expression across cell types of the developing cortex, but with an enrichment in progenitor cell types in the ventricular zones (Fig. 2*B*). To examine the spatiotemporal expression pattern of Tmem161b in vivo, we utilized a mouse model carrying the *Tmem161b* ^tm2b/+^ allele, which introduces a LacZ cassette in the *Tmem161b* locus ( *SI Appendix*, Fig. S2*C* ), that provides a spatiotemporal view of the expression of *Tmem161b* transcript in vivo. *Tmem161b*-driven ß-galactosidase activity was clearly enriched in the CNS at E12.5, with some expression in the heart (Fig. 2*C*), mirroring LacZ signal in enhancers that interact with the TSS of *TMEM161B*. In ferret, in situ hybridization for *Tmem161b* at postnatal day 2 (P2), an age at which neurogenesis and neuronal migration continue, mirrored mouse and human data with expression across most regions/cell types, with slight enrichment in the ventricular zone relative to the cortical plate (Fig. 2*D*). Together, these data demonstrate that *TMEM161B* is expressed in cell types relevant to cortical neurogenesis and folding across mice, ferret, and humans, and suggest that *TMEM161B* expression is developmentally regulated by several highly conserved CNS-specific enhancers.

### Tmem161b Null Embryos Show Defects Associated with Shh Signaling Disruption.

Mice homozygous for the *Tmem161b* ^tm2b^ allele (hereafter referred to as *Tmem161* KOs, or “KOs”) are *Tmem161b* null mice (*Tmem161b* ^−/−^) ( *SI Appendix*, Fig. S4*A* ) and died perinatally, but there was a normal Mendelian ratio until birth. The examination of homozygous mutant embryos revealed several gross abnormalities at E14.5 and E18.5, besides being smaller than their WT counterparts. They had a range of asymmetric eye defects, from coloboma to microphthalmia to frank anophthalmia (Fig. 2*E* and *SI Appendix*, Fig. S4*D* ). Midline defects ranged from minor cleft palate to failure of snout/facial development entirely, resulting in a proboscis-like structure (Fig. 2*E* and *SI Appendix*, Fig. S4*F* ).

Micro-CTs of several KO embryos ( *SI Appendix*, Fig. S4*F* ) confirmed the midline facial defects and additionally revealed holoprosencephaly, a malformation resulting from failure of separation of the dorsal forebrain into hemispheres (3). The examination of *Tmem161b* KO embryo brains at E14.5 and E18.5, confirmed this finding (Fig. 2*E* and *SI Appendix*, Fig. S4*E* ). The examination of more than 50 KO embryos showed that the eye and gross midline defects were completely penetrant, while ranging in severity and symmetry ( *SI Appendix*, Fig. S4 *D*–*F* ). The holoprosencephaly observed, like the other defects, was penetrant but variable; in some animals, it was noted as only involving the anterior ventral forebrain and in some there was a cyclopic appearance reminiscent of classic Shh mutants ( *SI Appendix*, Fig. S4 *C*–*F* ).

Holoprosencephaly, in combination with the craniofacial and eye defects, suggest a failure in patterning related to Shh signaling, but unlike *Shh* KO mice, *Tmem161b* KO mice lacked other classic Shh or ciliopathy phenotypes, specifically outside of the CNS (14, 29, 30). In all KOs examined, in gross (n > 50), as well as on microCTs (n = 8), no limb or digit abnormalities, renal cysts, or grossly apparent skeletal dysplasia were observed (29, 31). We did see previously described cardiac hypertrophy (16). Together, the gross phenotypes of the *Tmem161b* KO mouse imply a CNS-specific deficiency in normal Shh signaling, but no signs of Shh signaling defects or ciliopathy in tissues where developmental *Tmem161b* expression is low.

**Fig. 3.**
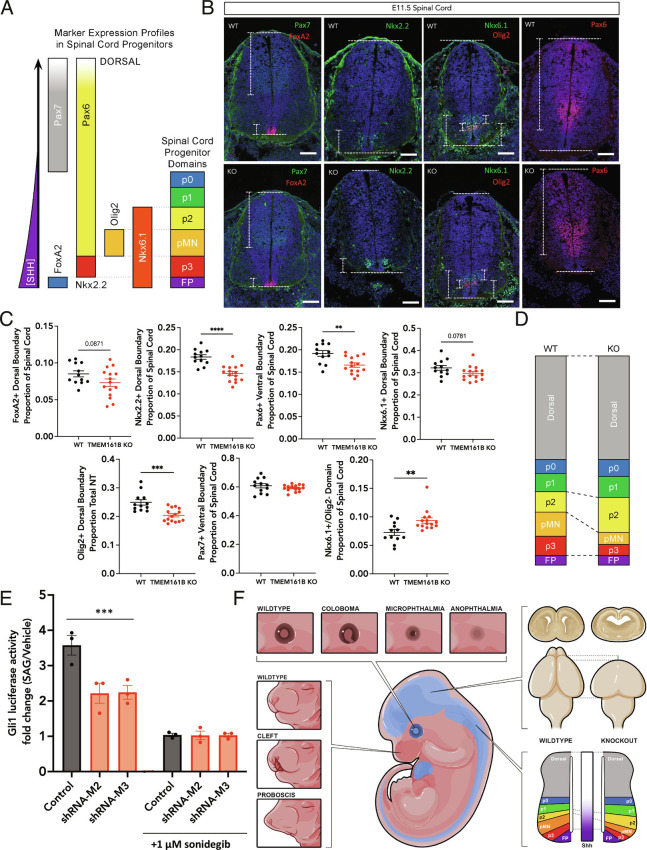
*Tmem161b* KO embryos show multiple sequalae of Shh disruption in the developing CNS. (*A*) Schematic of spinal cord markers examined in *Tmem161b* null embryos; in combination these markers define spinal cord domains in development patterned by response to a ventrally sourced Shh gradient. (*B*–*D*) *Tmem161b* KO mice show decreased ventral domains in the developing spinal cord. E11.5 control (n = 4) and *Tmem161b* KO (n = 5) embryos were harvested, and spinal cords including rostral and thoracic sections were prepared for IF for markers corresponding to ventral and dorsal progenitor domains in the spinal cord, which are dependent on a gradient of Shh signaling. (*C*–*E*) *Tmem161b* KO embryos showed a lower dorsal boundary of the Nkx2.2 domain/span of Nkx2.2 relative to the length of the spinal cord, with a correspondingly increased Pax6 domain, which is mutually exclusive with Nkx2.2 (*B*–*H* corrected multiple unpaired *t *tests of imaged fields, *P *< 0.0001 for Nkx2.2 and *P* = 0.0027 for Pax6 between WT and KO). There was a decrease in both dorsal boundary (*P* < 0.0001) and the span of the Olig2+ domain relative to total spinal cord length (*P* = 0.0324). Both FoxA2 and Nkx6.1 showed marginal decreases in their span in KOs relative to WTs (*P* = 0.0871 and 0.0781 respectively, *B*–*H* multiple testing corrected *t* tests), but the Nkx6.1+/Olig2− domain was increased in the KO relative to the WT (unpaired *t* test, *P* = 0.0064). There was no difference between Pax7+ domains in the WT vs. KO. Measurements of FoxA2, Nkx2.2, Pax6, Olig2, Pax7 and Nkx6.1 domains were performed by experimenters blinded to genotype. (*E*) *Tmem161b* supports sensitivity to Shh. SL2 cells (modified NIH3T3s with Gli1 luciferase reporter activity) were transfected with control or *Tmem161b* shRNAs and then treated with SAG for 48 h. Luciferase content was measured at the end of incubation to determine the relative amount of Gli1 transcriptional activity during the assay. Each data point represents an independent experimental replicate (separate passages of cells, between two batches of independently prepped plasmid and drug, and n = 3 technical replicates per condition/experiment). *Tmem161b* shRNA treated cells showed ~40% less Gli1 luciferase activity in response to SAG compared to controls (2- way ANOVA shows effect of shRNAs, *P* = 0.0048, effect of sonidegib, a smoothened antagonist, *P* < 0.0001, and interaction effect, *P* = 0.0054. Sidak post-hoc comparison, corrected, demonstrates *Tmem161b* shRNA treated conditions show less Gli1 luciferase activity relative to control shRNA, *P* = 0.0005 for shRNA-M2, and *P* = 0.0006 for shRNA-M3), and this effect was eliminated with the addition of sonidegib, suggesting that Tmem161b’s role in supporting Shh signaling is secondary to canonical Smoothened activation. (*F*) Summary of Shh related phenotypes observed across >50 *Tmem161b* KO embryos: eye defects ranging from coloboma to anophthalmia, craniofacial defects from minor cleft palate to failure of midline formation leading to proboscis, holoprosencephaly, and spinal cord patterning shifts. No limb abnormalities were noted across all nulls examined.

The characterization of *Tmem161b* mutant spinal cords confirmed patterning defects consistent with defective Shh signaling, as E11.5 KO animals showed a decrease in ventral spinal cord progenitor domains that require the highest levels of Shh signaling. The early developing spinal cord is patterned in part by a Shh gradient whose source is the notochord and subsequently the floorplate, where the most ventral domains require the highest Shh concentrations (Fig. 3*A*) (13, 32). Mutations that increase Shh activity cause the ventralization of the spinal cord at the expense of dorsal domains, usually marked by Pax6 and Pax7 (33), whereas mutations that abrogate Shh signaling result in the reverse (34). We performed IF in spinal cords of E11.5 *Tmem161b* KO and WT embryos with several markers (FoxA2, Nkx2.2, Olig2, Nkx6.1, Pax6, Pax7), which, in combination, provide information about spinal cord progenitor domains (Fig. 3*B*). We found decreased size of progenitor domains that require the highest Shh signal to pattern in *Tmem161b* KO embryos: a decrease in p3, marked by Nkx2.2+, with a corresponding increase in Pax6+, and a decrease in pMN, marked by Olig2+. We did not observe changes in the dorsal marker Pax7. Analysis of p2 (Nkx6.1+/Olig2−), a domain that is patterned by lower Shh activity than p3 and pMN revealed a significant expansion, suggesting a shift in identity from domains marked by the highest Shh activity to those with lower Shh activity (Fig. 3 *C* and *D*) We detected no change in Shh immunoreactivity itself, suggesting that *Tmem161b* functions in Shh-responsive cells, rather than causes loss of Shh secretion. The FoxA2+ domain showed a marginal decrease, but this floorplate marker occupies such a small region of the spinal cord that we lacked power to detect a domain shift on the scale of the Nkx2.2 and Olig2 decreases observed. When considered together (Fig. 3*D*), the domain marker shifts represent a spinal cord patterning change associated with altered Shh-mediated patterning in the *Tmem161b* KO embryos, though not a complete ablation of Shh signaling.

### Depletion of Tmem161b Impairs Transcriptional Response to Smoothened Agonist.

To directly examine Shh signaling in the presence or absence of *Tmem161b*, Gli1 transcriptional activity was measured in SL2 (Shh-Light II) cells exposed to Smoothened Agonist (SAG) either replete with *Tmem161b* or with *Tmem161b* knocked down using a validated *Tmem161b* shRNA ( *Methods, SI Appendix*, Fig. S4*B* ). *Tmem161b*-deficient cells demonstrated ~40% less Gli1 transcriptional activity than control cells following SAG treatment, and this effect was eliminated by blocking Smoothened activation. This suggests that *Tmem161b* promotes normal sensitivity to Shh ligand, and that this support is dependent on Smoothened activation. There was no effect of *Tmem161b* level on Gli1 readout without stimulation, suggesting that the lack of Tmem161b does not cause a change in the baseline levels of Gli1 transcription (Fig. 3*E*). Altogether, the *Tmem161b KO* mouse model and these in vitro data, support a role for *TMEM161B* in supporting normal Shh signaling in the developing CNS (Fig. 3*F*).

### Disruption of Tmem161b In Vivo Alters Neuronal Positioning in Mice.

**Fig. 4.**
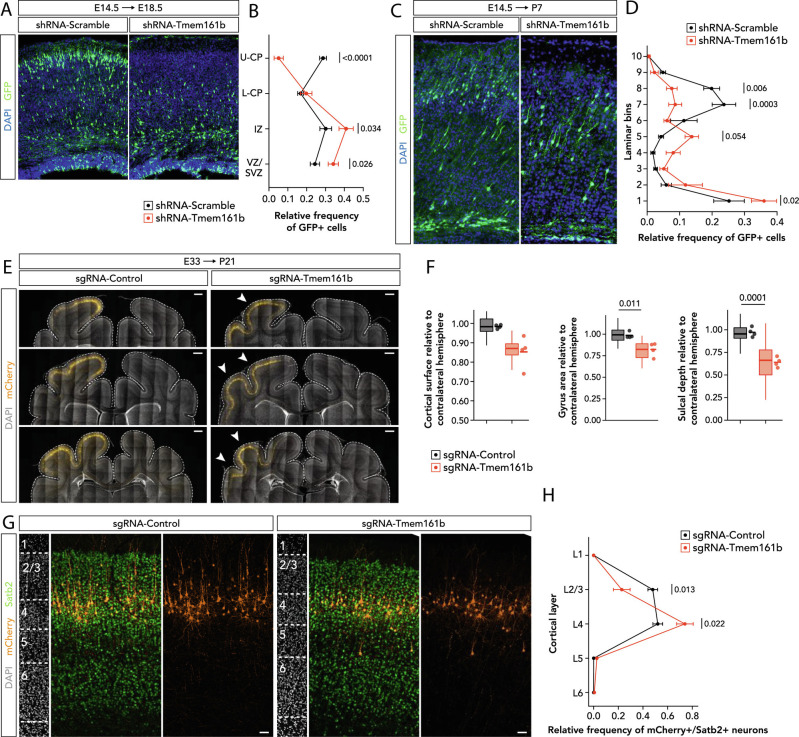
In vivo disruption of Tmem161b causes abnormal cortical histogenesis. (*A*–*D*) Knockdown of *Tmem161b* in the developing cortex causes cell autonomous neuronal positioning defects. (*A*, *B*) CD1 embryos were electroporated at E14.5 with either a control plasmid expressing a non-targeting shRNA (n = 6), or an shRNA targeting mouse *Tmem161b* (n = 6); both plasmids had second ORF containing a GFP tag to visualize electroporated cells. Cells electroporated with an shRNA targeting *Tmem161b* showed a cell autonomous lamination defect, concentrating more in the VZ/SVZ and IZ than in the cortical plate after the 4-d period between electroporation and harvest; of the *Tmem161b* depleted cells that did enter the cortical plate, virtually none migrated into the upper cortical plate by the analysis timepoint. 2-way ANOVA of plasmid condition X cortical region showed an interaction effect, *P* < 0.001, with post Sidak multiple comparison tests, corrected for multiple testing, demonstrating more knockdown cells found in the VZ/SVZ (*P* = 0.0255) and IZ (*P* = 0.0336), and more control cells found in the upper cortical plate (*P* < 0.0001). >200 GFP+ cells per brain were analyzed. (*C*, *D*) IUE performed as described above, but electroporation at E14.5, and analysis at P7 (i.e., longer survival), where final distribution of GFP+ cells was quantified across 10 laminar bins of the P7 cortex divided evenly from pial surface to bottom of cortex. Knockdown of *Tmem161b* at E14.5 led to an altered distribution of GFP+ cells at P7 (2-way ANOVA of plasmid condition X cortical region showed an interaction effect, *P* < 0.001, with post Sidak multiple comparison tests, corrected for multiple testing). (*E*–*H*) *Tmem161b* knockdown via IUE of pyramidal cell progenitors in the developing ferret cortex at E33.5 using sgRNA targeting *Tmem161b* results in reduced cortical surface, reduced gyrus size and sulcal depth, and altered neuronal positioning in the postnatal ferret cortex at P21. (*E*) Coronal sections of through the P21 ferret cortex processed for immunohistochemistry against mCherry (orange) counterstained with DAPI (gray) in control and *Tmem161b*-knockdown ferrets. Dotted lines indicate the dorsal surface of the cerebral cortex. Arrows indicate the reduced size of gyri and sulci in somatosensory cortical areas with neurons electroporated with the sgRNA-Tmem161b plasmid. (*F*) Quantification of the cortical surface, gyrus size, and sulcal depth in control and *Tmem161b*-knockdown ferret brains. For each gross anatomical variable, the ratio between the region with electroporated cells and the corresponding region in the contralateral, non-electroporated hemisphere was calculated. Four ferret brains per experimental condition and >10 coronal sections per brain were analyzed for each variable (n = 52 sections in sgRNA-Control, n = 52 sections for sgRNA-Tmem161b). Two-tailed Student’s *t* test: *P* = 0.020 for cortical surface, *P* = 0.011 for gyrus size, and *P* = 0.0001 for sulcal depth. (*G*) Coronal sections through the somatosensory cortex processed for immunohistochemistry against Satb2 (green) and mCherry (orange) counterstained with DAPI (gray) in control and *Tmem161b*-knockdown ferrets at P21. Confocal images illustrating the distribution of mCherry+/Satb2+ neurons and quantified across cortical layers. Note the presence of some mCherry+/Satb+ neurons in L5, which were rarely seen in the control brains. (*H*) Relative frequency of the distribution of mCherry+/Satb2+ neurons quantified across cortical layers of P21 ferret cortex. Knockdown of *Tmem161b* leads to an altered distribution of mCherry+/Satb2+ cells in L2/3 and L4 (two-way ANOVA, *P* < 0.001, with post Sidak multiple comparison tests). Four ferret brains per experimental condition and >200 cells per brain were analyzed. Data are shown as mean ± SEM. Data shown as box plots represent the distributions of values from all coronal sections quantified per condition, and the adjacent data points and lines represent the averages per ferret and averaged mean per condition, respectively*.* (Scale bars, 1 mm (*E*) and 50 μm (*G*).)

To determine whether Tmem161b plays a cell-autonomous role in neuronal differentiation and lamination that could underlie the PMG seen in humans, we performed in utero electroporation (IUE) in the developing cortex on E14.5 embryos using a validated *Tmem161b* shRNA ( *Methods, SI Appendix*, Fig. S4*B* ) and collected E18.5 embryos for examination. Cells depleted of *Tmem161b* showed abnormal cortical positioning; many failed to enter the cortical plate entirely after exit from the ventricular zone, and if they did, they did not reach the upper cortical plate, as observed for control cells (Fig. 4 *A* and *B*). Extending similar experiments to a P7 survival confirmed that the observed defects were not simply a developmental delay (Fig. 4 *C* and *D*) and showed that *Tmem161b* knockdown altered the position of the progeny of electroporated cells at E14.5, biasing them to lower cortical layers, leading to fewer total GFP+ cells overall, and lower GFP+/Satb2+ double positivity ( *SI Appendix*, Fig. S6). Although the abnormal positioning could reflect defective migration per se, the loss of Satb2 staining, a marker biased to upper cortical layers, suggests a cell fate shift of upper layer neurons towards lower layer fates.

### Tmem161b Loss-of-Function Results in Reduced Gyri and Altered Neuronal Positioning.

To determine whether TMEM161B regulates the development of cortical gyri specifically, we performed parallel IUE experiments in the ferret, a gyrencephalic species. We electroporated ferret embryo brains with plasmids encoding single-guide RNA targeting Tmem161b (sgRNA-Tmem161b) or control plasmid at embryonic day E33 ( *SI Appendix*, Fig. S5), corresponding to the time of generation of upper-layer neurons (35), using standard procedures (8, 36). Ferret brains were collected at postnatal day 21 and sectioned coronally ( *Methods, SI Appendix*, Fig. S5). Large numbers of electroporated cells were evident microscopically in roughly corresponding regions of sensory-motor cortex in both control and experimental conditions. We first analyzed three gross anatomical features (dorsal cortical surface, gyrus size, and sulcus depth) by quantitatively assessing the region of electroporated cells relative to the non-electroporated region in the contralateral hemisphere of coronal brain sections, following published methods (8). We found a significant decrease in the surface of the dorsal cortex in *Tmem161b*-knockdown ferrets compared to controls (Fig. 4 *E* and *F*). Similarly, gyri and sulci of knockdown brains were significantly smaller in cortical regions with successfully electroporated cells (Fig. 4 *E* and *F*). These results suggest that Tmem161b is essential for the normal development of gyri in the ferret cortex.

To examine the cytoarchitectonic organization of the cortex in Tmem161b-knockdown ferrets, we next performed immunohistochemistry for Satb2, a transcription factor which is expressed in callosal projection neurons and is highly enriched in the upper layers of the cortex. We quantified the relative distribution of Satb2-expressing electroporated regions across the cortical column and found a modest, but significant, reduction in the proportions of Satb2+/mCherry+ neurons in layers 2-3 and 4 (Fig. 4 *G* and *H*). These results are consistent with the altered neuronal positioning in *Tmem161b* knockdown experiments in mice analyzed at P7. As in the mouse, the ferret results are most easily interpreted as a reflecting premature withdrawal of progenitors toward precocious deep-layer neuronal fates, though additional cell type-specific markers were not available in ferret to further address this. Altogether, our data indicate that *Tmem161b* loss-of-function in vivo leads to disruption of cortical development both in mice and ferrets and confirms that disrupting *Tmem161b* can lead to gyral abnormalities relevant to PMG as seen in the human individuals described above.

### Tmem161b Null Embryos Have Abnormal Primary Cilia.

**Fig. 5.**
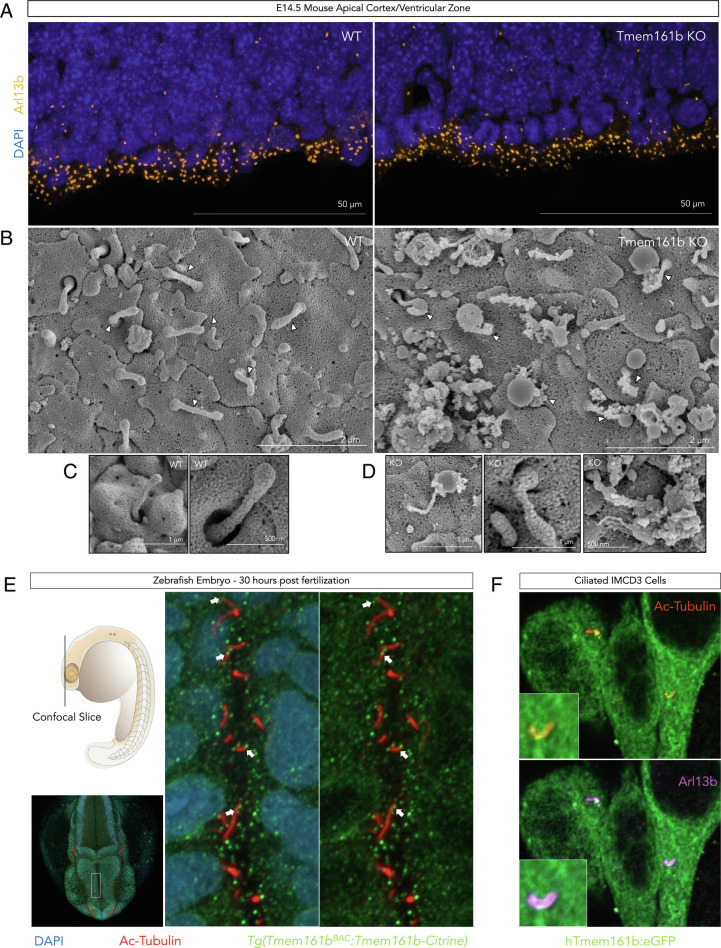
*Tmem161b* null embryos have abnormal primary cilia in the developing CNS, and TMEM161B protein localizes to primary cilia. (*A*) *Tmem161b* KO embryos show normal numbers of apical primary cilia in the cortex. N = 7 WT and N = 8 *Tmem161b* KO embryos had cortices stained with Arl13b, and number of apical puncta were counted. There was no difference in the number of puncta between genotypes, but a qualitative appearance of more rounded/abnormally shaped puncta was noted leading to follow-up SEM. (*B*) *Tmem161b* KO apical primary cilia show structural abnormalities. SEM images of the apical cortical surface of *Tmem161b* KO mice and control with some primary cilia labeled with white arrows. WT primary cilia display normal morphology, while the KO surface shows increased vesicular debris, and primary cilia with ballooned tips and disrupted stalks. (*C*, *D*) Examples of *Tmem161b* KO primary cilia. Typical examples of the abnormalities found in *Tmem161b* KO primary cilia – ciliary tip ballooning or shortened cilia with abnormal contour. (*E*) Tmem161b localizes to primary cilia in zebrafish hindbrain. Confocal imaging of transgenic zebrafish that express a citrine tagged Tmem161b at 30 h post fertilization shows co-localization with citrine puncta and primary cilia marker Acetylated tubulin in the hindbrain ventricular zone; see also Movie S1. (*F*) TMEM161B localizes to primary cilia in IMCD3 cells. GFP tagged human TMEM161B was expressed in IMCD3 cells, which were then ciliated and confocal imaging performed. GFP signal showed clear co-localization with primary cilia markers Arl13b and Acetylated tubulin, see also Movie S2.

To explore the basis of the *Tmem161b* related Shh signaling defect, we examined apical primary cilia of radial glial cells at the ventricular surface in E14.5 *Tmem161b* KO mice and found widespread and significant abnormalities of structure. *Tmem161b* KO embryos showed no differences in the number of *Arl13b*+ cilia counted overall, although the puncta appeared smaller and with differing circularity, suggesting malformed primary cilia (Fig. 5 *A* and *B* and *SI Appendix*, Fig. S7*A* and *Methods* ). Scanning electron microscopy (SEM) of the same ventricular surface (Fig. 5*B*) confirmed that many *Tmem161b* KO primary cilia are malformed, being more tapered or stub-like (Fig. 5 *B*–*D*). More dramatically, SEM of the *Tmem161b* KO cortical apical membrane revealed large amounts of membranous debris, very little of which was seen in WT specimens (Fig. 5*B* and *SI Appendix*, Fig. S7*B* ). Most strikingly, a proportion of the KO cilia showed ciliary tip ballooning, with vesicular debris often directly in contact with ciliary tips (Fig. 5*D* and *SI Appendix*, Fig. S7*B* ). Overall, SEM of *Tmem161b* KO mice demonstrates a range in ciliary abnormalities characterized by ciliary shortening, ciliary tip ballooning, and vesicular debris.

### Tmem161b Localizes in Part in Primary Cilia.

Based on the abnormal primary ciliary structure described, we hypothesized that Tmem161b protein may localize, at least in part, to the primary cilium. Although several primary antibodies failed to produce sufficient signal for experimentation, we examined a zebrafish transgenic line with a citrine tagged Tmem161b [Tg(Tmem161b^BAC^:Tmem161b-citrine)] (16), and also expressed GFP-tagged human TMEM161B in IMCD3 cells (mouse medullary collecting duct cells, utilized for studies of primary cilia) to examine subcellular localization of Tmem161b. In the transgenic zebrafish, citrine puncta co-localized with primary ciliary marker acetylated tubulin in the hindbrain ventricle (Fig. 5*E* and Movie S1). In culture, IMCD3 cells expressing human TMEM161B-GFP showed co-localization with primary ciliary markers Arl13b and acetylated tubulin (Fig. 5*F* and Movie S2). The independent identification of primary ciliary localization of Tmem161b in both zebrafish in vivo as well as with human TMEM161B in mammalian cells in vitro shows TMEM161B localizing, though non-exclusively, to primary cilia, with the remainder of subcellular localization signal in the ER or across diffuse membranes.

## Discussion

Here we show that *TMEM161B* belongs to an evolutionarily ancient superfamily of proteins with orthologs found in plants and even single-celled eukaryotes and that it has essential function in nervous system development at least two stages—early midline patterning and later neuronal organization. Biallelic deleterious variants in *TMEM161B* disrupt cortical gyration in humans, mirrored by the results of partial knockdown of *Tmem161b* disturbing normal cortical lamination in mice and normal gyration in ferrets. Complete loss of *Tmem161b* in mouse leads to a more severe earlier patterning phenotype—holoprosencephaly and spinal cord patterning defects consistent with loss of Shh signaling. Confirming this, disrupting *Tmem161b* impairs cellular response to stimulation by SAG. Finally, a portion of Tmem161b is found in the primary cilium, and loss of Tmem161b leads to dramatic primary cilia abnormalities that may underlie the phenotypes associated with its disruption across multiple models and time points in development.

The human syndrome caused by biallelic mutations in *TMEM161B* is stereotyped in its presentation of diffuse PMG, intellectual disability, and seizures with limited extra-CNS disease. The accompanying manuscript by Wang et al. reports an additional four families to the three in this report with the same clinical description. Also of interest, a recent GWAS for cerebral cortical sulcal depth in humans identified a genome-wide significant SNP (rs304136C; chr5:88170066) that is located slightly upstream of *TMEM161B* and close to the enhancers highlighted here, suggesting that both rare and common *TMEM161B* variants may regulate cerebral cortical gyration (37). The converging rare and common genetics in humans, and strong evidence of essential roles in mammalian models, suggests that *TMEM161B* may be a central regulator of cerebral cortical folding.

The connection of *TMEM161B* to primary cilia structure in our work may reconcile the differing cortical gyration phenotypes of human individuals and “early” patterning phenotype of the null mouse model described. PMG and holoprosencephaly represent points on the spectrum of severity of ciliary phenotypes in other disorders. PMG is a common manifestation of primary ciliopathies, such as those associated with *TMEM216, TCTN1,* and *BBS1-10* (12, 15, 38, 39), while severe ciliopathies in humans, such as Meckel syndrome, can also lead to midline defects including frank holoprosencephaly (40), though holoprosencephaly is typically caused by pathogenic variants in genes encoding SHH signaling components (5, 41).

One plausible explanation as to why the humans with *TMEM161B* missense variants have polymicrogyria without gross midline defects is that they have sufficient TMEM161B function to promote normal forebrain patterning but insufficient function for proper neuronal proliferation, migration, and gyrification (i.e., a moderate ciliary phenotype from partial loss of function missense variants). On the other hand, the mouse null models, lacking Tmem161b function entirely, show manifestations of an early or more severe ciliopathy affecting overall forebrain patterning. In Wang et al.’s accompanying report, knock-in mouse models of patient missense alleles demonstrate cortical plate lamination defects without forebrain patterning defects, further supporting this interpretation. Future work determining the cellular role of TMEM161B, and clear functional assays of that role, will allow more characterization of partial LoF alleles versus full null alleles ( *SI Appendix*, Discussion).

Recent experimental work in ferret directly implicated Shh signaling in normal cortical folding through the maintenance of HOPX+ outer radial glial cells (oRGs), which are more common in gyrencephalic mammals (9, 10, 42, 43) and accumulate in prospective gyral regions compared to their HOPX- counterparts (8, 44). Shh activity specifically suppresses differentiation of HOPX+ oRGs; in utero electroporation-based suppression of Shh using a competitive inhibitor of Shh [HhipΔC22 (45)] decreases the number HOPX+ oRGs and local cortical folding. Thus modifying Shh signaling is sufficient to impact local gyrification through influencing oRGs whose proliferation is associated with gyral formation (8). Our connection of *Tmem161b* to Shh signaling, and ferret IUE experiments are consistent with this model, and our ferret findings in particular are reminiscent of the effects of ectopic local HhipΔC22 expression, as both lead specific disruption of layer II/III relative to lower cortical layers and lead to local decrease in sulcal depth and gyral size. The mechanism of this shift may reflect loss of Shh-mediated suppression of oRG differentiation, which would cause an early termination of neurogenesis and lead to the upper layer-specific deficit seen. In this context, our results, including the observation that *TMEM161B* is enriched in *HOPX*+ oRGs in human fetal brain (among other oRGs), suggest that decreased sensitivity to Shh in a developing gyrencephalic cortex deficient in *TMEM161B* could lead to abnormal gyration/polymicrogyria by changing the behavior of gyrencephalic oRGs and altering neuronal proliferation and lamination.

The lack of observed ciliopathy or Shh-related phenotypes outside the brain (e.g., renal cysts, skeletal dysplasia, or limb and digit abnormalities) in our KO mice suggests specific importance of *TMEM161B* expression in the developing CNS, or that the developing CNS could be specifically vulnerable to a loss of TMEM161B in a way other tissues or developmental stages are not. Specialized proteins supporting ciliary structure or Shh in specific tissues is not unprecedented. For example, the phosphatase Eya1 is involved in supporting Shh in the eye and cerebellum but is not required in the developing spinal cord (46). Beyond Shh, the primary cilium is a key hub for other signaling pathways including TGF-beta-mediated signaling (47), Wnt signaling (48, 49), and signaling by many ciliary GPCRs (50, 51). Therefore, the impact of TMEM161B depletion may depend on the cellular context in which ciliary signaling pathways are most vulnerable to disruption for those cells. Given the ubiquitous expression of *TMEM161B* across many tissues after fetal development and the many cell-type-specific roles of the primary cilium (with cell-type-specific complements of ciliary GPCRs), we anticipate future characterization of *TMEM161B* function in multiple tissues, such as in the heart or kidney, and will help clarify the mechanisms by which this protein modulates cellular signaling.

## Methods

### Human Subjects.

Subjects were identified and evaluated in clinical settings, and biological samples were collected for research purposes after obtaining written informed consent. All protocols were approved by local institutional review boards of King Faisal Specialist Hospital and Research Center (RAC number: 2080006), NIH (IRB approval number: 15HG0130), and Boston Children’s Hospital (protocol: 05-05-076R). For human genetics sequencing information, see *SI Appendix, Methods* .

### Mammal Maintenance.

For animal generation details, see *SI Appendix, Methods* . All experiments were approved by institutional animal use committees specific to where experiments were conducted (Harwell, or Boston Children’s Hospital). All mice for analysis were collected during embryonic ages due to early lethality of the homozygous mutant animals. *Tmem161b^tm2b/WT^* (these are effectively *Tmem161b +/−,* or *Tmem161b* heterozygous mice) mice were bred in heterozygote-heterozygote pairings to generate both WT and *Tmem161b^tm2b/tm2b^* [these are effectively *Tmem161b −/−* (null)] embryos used in experiments. As *Tmem161b* null mice die at birth, all experiments with null mice were conducted via prenatal harvest after timed pregnancy, and all experiments utilized matched control WT littermates for comparison. For details on LacZ preparation of *Tmem161b^tm2b/WT^* animals, see *SI Appendix, Methods* . Ferrets (Mustela putorius furo) were obtained from Marshall Bioresources and animals were same-sex housed within a dedicated Large Animal Facility of Boston Childrens Hospital on a 12-h light/dark cycle at 18 °C to 23 °C, with rotating sensory enrichment. Food and water were available ad libitum.

### In Situ Hybridization/RNAscope.

The RNAscope Multiplex Fluorescent V2 was performed according to the manufacturer’s protocol. Tissue is harvested E14.5 for mouse and P2 for ferret. The tissue slides were collected by cryosection for mouse and paraffin section for ferret. After removing OCT (Optimal cutting temperature compound) or paraffin, the section was baked for 30 min on 50 °C hot pplate, followed by antigen retrieval (10 min at 100 °C) and treated by Protease plus for 30 min at 40 °C. Each Probe was hybridized for 2 h at 40 °C, washed, and hybridized with C1 or C2 target-binding amplifiers allowing for signal amplification of single RNA transcripts. The signal was detected with Opal 620 fluorephores. The sections were counterstained with DAPI, then mounted/coverslipped with ProLong Glass Antifade Mountant (Thermofisher).

### Mouse In Utero Electroporation.

In utero electroporation was performed as described previously (52); for this experiment, we electroporated CD1 mouse embryos at E14.5 with either a control shRNA (scrambled) or shRNA-TMEM161B-M2 and harvested at E18.5 or P7. After overnight 4% PFA fixation and cryo-sectioning, GFP+ signal was amplified via immunofluorescence (see below), and GFP+ cells were counted in representative sections across the entire GFP+ region of the cortex of each embryo brain examined. N = 6 total embryos from at least 2 separate electroporations were counted in each condition, and GFP+ cells were binned according to their migratory position for analysis; the bins were determined via examination of the DAPI signal to defined embryological regions: ventricular zone/subventricular zone (VZ/SVZ), intermediate zone (IZ), infragranular cortical layers/lower cortical plate (L-CP), and upper cortical plate (U-CP). Cell counts were performed blinded to plasmid condition. P7 harvested embryos were quantified using 10 laminar bins evenly divided between the pial surface and the bottom of the cortex; comparisons were conducted with multiple discovery correction. For mouse IF experimental details, see *SI Appendix, Methods* .

### Ferret In Utero Electroporation.

Ferrets were maintained on the operation table under a constant 3% isoflurane flow using a nose mask. After laparotomy, the uterus was exposed and embryos were injected intraventricularly with 1.5 to 3 μL of a solution containing 0.1% Fast Green (Sigma) in sterile PBS and 1 μg/μL of either sgRNA-Tmem161b vector or sgRNA-control vector, together with 1 μg/μL of pCAG-mCherry vector encoding a fluorescent reporter protein. Following intraventricular injections, five 50-ms electric pulses of 150 V were delivered at 1 s interval using sterile forceps-shaped electrodes connected to an electroporator (BTX ECM 830). The electrodes were positioned to target pyramidal cell progenitors in the subventricular zone of the dorsal cortex. Ferrets were monitored until they woke up and underwent postoperative care (daily 0.2 mg/kg meloxicam given every 24 h for 72 h). For details of ferret histology methods, see *SI Appendix, Methods* .

### Gli1 Luciferase Assay.

Shh-Light II (SL2) cells were obtained from American Type Culture Collection and cultured according to their recommendations. SL2 cells were plated in 6-well plates, then each well was transfected with a 2,500 ng single shRNA using Lipofectamine 3000 (Thermo Fisher), either with a scrambled, non-targeting shRNA, or shRNA TMEM161B-M2 or shRNA TMEM161B-M3. Cells were re-plated 15 to 16 h after transfection in 96-well plates in DMEM with 0.5% calf serum. After 24 h of transfection, the cells were stimulated with 500 nM SAG or vehicle (DMSO) and treated with 1 μM of Sonidegib or vehicle simultaneously in DMEM with 0.5% calf serum for 48 h. Transfection efficiency was estimated via fluorescent tag at ~60% for each experiment The cells were then lysed and tested using dual-luciferase reporter assay system (Promega). Replicates were completed on separate plates each with respective controls. We then performed a separate qPCR validation of the shRNAs used after the SL2 assays in extra wells transfected in the same manner as those used in the luciferase assay as described above.

### Scanning Electron Microscopy.

Embryos were harvested at E14.5 and dissected in PBS to isolate brains, in which incisions were made to expose the ventricular surface. Brains were immersion fixed with warmed 2.5% Glutaraldehyde/2.0% Paraformaldehyde in Sodium Cacodylate buffer, pH 7.4 (Electron Microscopy Sciences). After fixation, the ventricular surface was isolated through further dissection, and pieces of interest were subsequently dehydrated through ethanol, and critical point dried (Samdri-PVT-3D, Tousimis, Inc.) using liquid CO_2_. Dehydrated tissue pieces were mounted on aluminum stubs using carbon tabs (Electron Microscopy Sciences). Samples were sputter coated to a 20 nm platinum coating (Cressington 208HR SN C4106 sputter coater, Cressington Scientific instruments UK, Watford WD19 4BX, England), and then imaged on a Hitachi S-4800 scanning electron microscope (Hitachi, Ltd.). SEM was performed on embryos from four independent litters, which included a total n = 9 KO animals and n = 5 WT controls examined.

### Zebrafish Immunofluorescence.

Embryos were euthanised in Tricaine and immediately fixed in 4% PFA + PBS overnight at 4 °C. Following fixation, embryos were washed ×3 in PBST (PBS + 0.1% Triton X-100) and permeabilized in 1% Triton X-100 + PBS for 8 h. The embryos were then blocked in 0.5% Triton X-100 + 10% Goat Serum in PBS for 1 h and incubated in primary antibodies (GFP 1:1,000 (AbCam Cat# ab13970) and Acetylated Tubulin 1:500 (Sigma Cat# T6793) overnight at 4 °C. The embryos were then washed 3× in PBST followed by incubation in secondary antibodies (Alexa Fluor 488/594 1:500) overnight at 4 °C. The embryos were then incubated in DAPI for 20 min, washed 3× in PBST and mounted in 1% agarose for imaging. Immunofluorescent images were taken on a Zeiss LSM900 using Airyscan with either 40× water immersion or 63× oil immersion objectives. Images acquired were then processed using either Zen (Zeiss), IMARIS (Bitplane) and ImageJ/FIJI.

### IMCD3 Cell Culture.

IMCD3 cell were grown in DMEM:F12 + glutamax (Gibco Cat# 10565018) supplemented with 10% FBS + 1% Penicillin/ Streptomycin (P/S). Cells were transfected with hTmem161b-eGFP plasmid using Lipofectamine 3000 Transfection Reagent (Invitrogen Cat# L3000008) according to the manufacturer’s instructions. 48 h after transfection cells were washed ×2 with PBS and incubated with serum starvation media (DMEM:F12 + glutamax + 1% P/S) for 24 h. After serum starvation, cells were washed with PBS and serum re-stimulated in growth media for 1 min. For imaging details, see *SI Appendix, Methods* .

## Supplementary Material

Appendix 01 (PDF)

Dataset S01 (DOCX)

Movie S1. **Related to Figure 5E** XZ Plane video of inset region showed in Figure 5E. Green signal corresponds to Tmem161b-citrine, and red signal to Acetylated tubulin. Note the green signal as puncta at multiple ciliary tips as well as ciliary bases.

Movie S2. **Related to Figure 5F** Video of z-stack from maximum intensity projection displayed in Figure 5F of ciliated IMCD3 cells with transfected humanized TMEM161B-GFP, co-stained for Acetylated tubulin. Green signal corresponds to Tmem161B-GFP fusion protein, and red signal to Acetylated tubulin. Note accumulation of GFP+ signal at ciliary tips in this field.

## Data Availability

All study data are included in the article and/or *SI Appendix* .
