# Supplementary material for: TMEM161B regulates cerebral cortical gyration, Sonic Hedgehog signaling, and ciliary structure in the developing central nervous system
Source: Proc Natl Acad Sci U S A. 2023 Jan 20;120(4):e2209964120. doi: 10.1073/pnas.2209964120 (PMC9942790; doi:10.1073/pnas.2209964120)
Supplement: Supplementary file 1 — Appendix 01 (PDF) [file pnas.2209964120.sapp.pdf]

TMEM161B regulates cerebral cortical gyration, Sonic Hedgehog signaling, and ciliary structure in the developing central nervous system

SK Akula et al.

Supplementary Information

1. Supplementary Figures 1-7, Information for Supplementary Videos 1-2
2. Clinical Case Summaries of Individuals with biallelic *TMEM161B* variants
3. Supplementary Discussion
4. Supplementary Methods

Supplementary Figures

*Figure legends follow each figure on a separate page

A

Modeled effects of identified variants on predicted TMEM161B structure

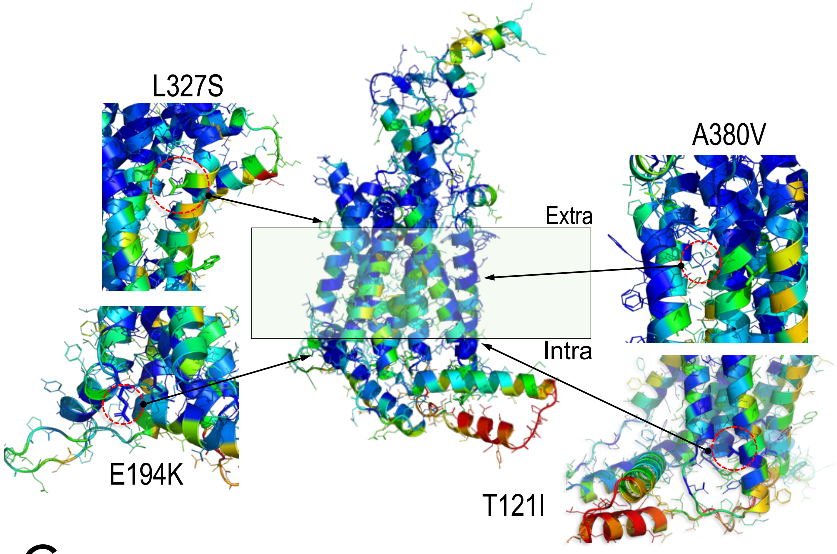

B

Post-mortem histology from cortex of Individual BFP906 displaying diffuse PMG

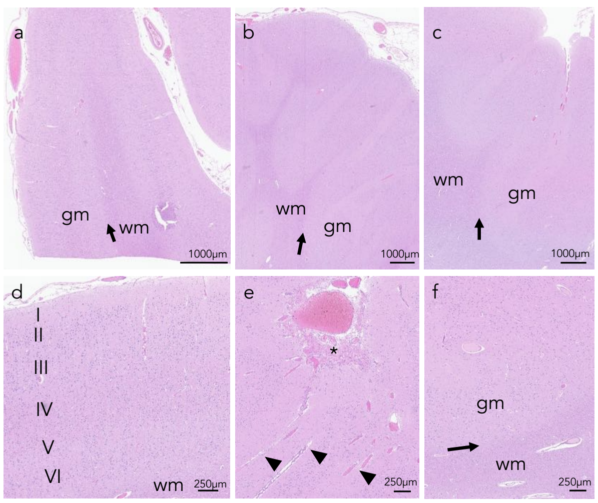

C

Conservation of amino acids altered by identified *TMEM161B* missense variants reported in vertebrate species

Thr121Ile

TMEM161B Orthologs	AAs 114-128
Human	ATVVYLVTEVYYNFM
Chimpanzee	ATVVYLVTEVYYNFM
Rhesus	ATVVYLVTEVYYNFM
Mouse	ATIVYLVTEVYYSFM
Rat	ATVVYLVTEVYYSFM
Cat	ATLVYLVTEVYYNFM
Ferret	ATVVYLVTEVYYNFM
Opossum	ATVVYLVTEVYYSLM
Chicken	ATVVYVVTEAYYSIV
Sea Turtle	ATVVYLVTEAYYSLM
Xenopus	ATVVYLVTEAYYSLV
Zebrafish	ATVVYLITELYFCVA
Lamprey	ATLVYVLTEAYYTLA

Glu194Lys

TMEM161B Orthologs	AAs 186-200
Human	LIVTENYLEFGLETG
Chimpanzee	LIVTENYLEFGLETG
Rhesus	LIVTENYLEFGLETE
Mouse	LIVTENYLEFGLETG
Rat	LIVTENYLEFGLETG
Cat	LIVTENYLEFGLETG
Ferret	LIVTENYLEFGLETG
Opossum	LIVTENYLEFGLETG
Chicken	LIVTENYLEFGLESG
Sea Turtle	LIVTENYLEFGLESG
Xenopus	LIVTENYLEFGLETG
Zebrafish	LIVTENYLEFGLETG
Lamprey	LIVSERFLEFGLDEG

Ala380Val

TMEM161B Orthologs	AAs 373-387
Human	VIALQYVAPLVMLLH
Chimpanzee	VIALQYVAPLVMLLH
Rhesus	VIALQYVAPLVMLLH
Mouse	VIALQYVAPLVMLLH
Rat	VIALQYVAPLVMLLH
Cat	VIALQYVAPLVMLLH
Ferret	VIALQYVAPLVMLLH
Opossum	VIALQYVAPLVMLLH
Chicken	VIALQYVAPLVMLLH
Sea Turtle	VIALQYVAPLVMLLH
Xenopus	VIALQYMAPLVMLLH
Zebrafish	VIALQYIAPLVMLLH
Lamprey	VIALQYLAPLVLLLN

Leu327Ser

TMEM161B Orthologs	AAs 321-335
Human	LLCALRLAMMRSHLQ
Chimpanzee	LLCALRLAMMRSHLQ
Rhesus	LLCALWLAMMCSHLQ
Mouse	LLCVLRLAMMRSHLQ
Rat	LLCVLRLAMMRSHLQ
Cat	LLCALRLAMMRSHLQ
Ferret	LLCALRLAMMRSHLQ
Opossum	LLCALRLALMRNHLQ
Chicken	LLCALRLAMMRSHLQ
Sea Turtle	LLCALRLAMMRKHLQ
Xenopus	VLCALRLAMMRSHLQ
Zebrafish	LLCVLRLAMMRHHLQ
Lamprey	LLCVLRLLLTRSHLQ

D

Validation of splicing variant identified in UDN172478

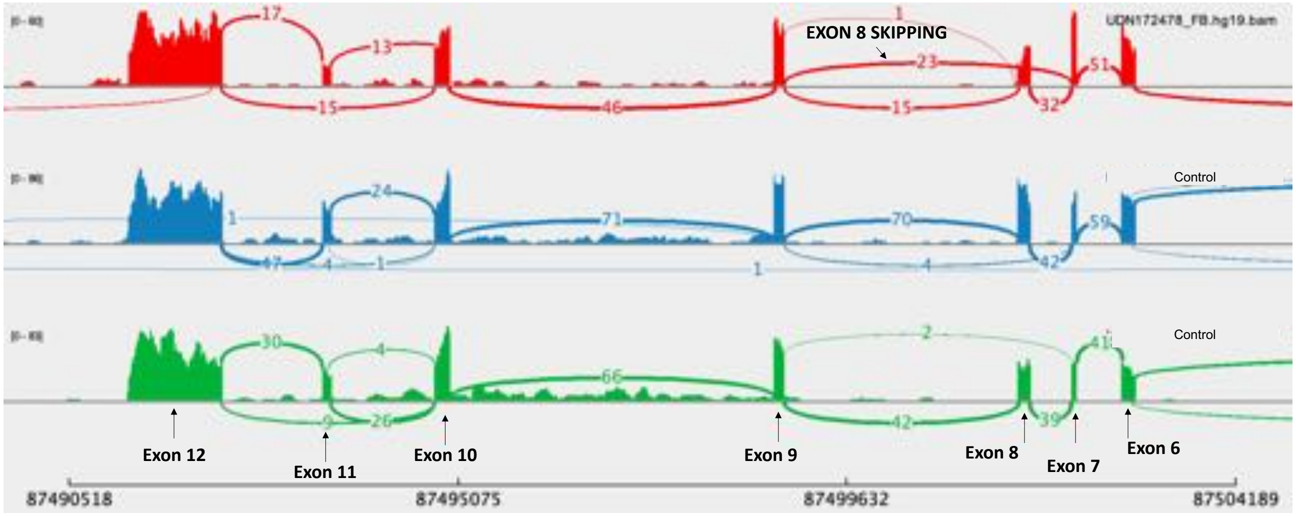

Figure S1: Related to Figure 1

(A) Predicted structural consequences of *TMEM161B* variants. The identified missense variants are all predicted to cause disruption to highly conserved residues in TMEM161B, either disrupting core packing of TM helices (p.Thr121Ile, p.Leu327Ser, p.Ala380Val) or cause charge reversal in another highly conserved pocket (p.Glu194Lys).

(B) Histologic findings in BFP906 from post-mortem neuropathologic examination. Medial occipital region showing normal 6-layer neocortex (a) adjacent to polymicrogyria (b). Note the well-defined white (wm) matter-grey matter (gm) junction (arrows). Polymicrogyria was extensive throughout both lobes (c, example from frontal lobe). High magnification from the normal appearing occipital cortex reveals a well-organized 6-layer cortex (I-VI). Fusion of the molecular layer with entrapment of blood vessels is seen extensively in the abnormal cortex (e; arrowheads) and leptomeningeal neuroglial heterotopia (e; asterisk) is seen focally at the surface of the brain, as is classical for polymicrogyria. The polymicrogyric cortex is thinned with reduced numbers of neurons and shows poor lamination and the well-defined white matter-grey matter junction (f).

(C) Evolutionary conservation of *TMEM161B* variants identified. All identified missense variants (marked in Red) occur in residues entirely conserved in vertebrates.

(D) Validation of splice-region variant identified in Family B. RNA-sequencing from fibroblasts derived from UDN172478, and unaffected controls demonstrates that the splicing variant c.800+5G>A [chr5(GRCh37):g.87501626C>T] causes significant exon skipping of exon 8 that disrupts TM5 and the highly conserved binding pocket.

A **Analysis of human plac-seq Data showing conserved active fetal brain enhancers interacting with TMEM161B TSS**

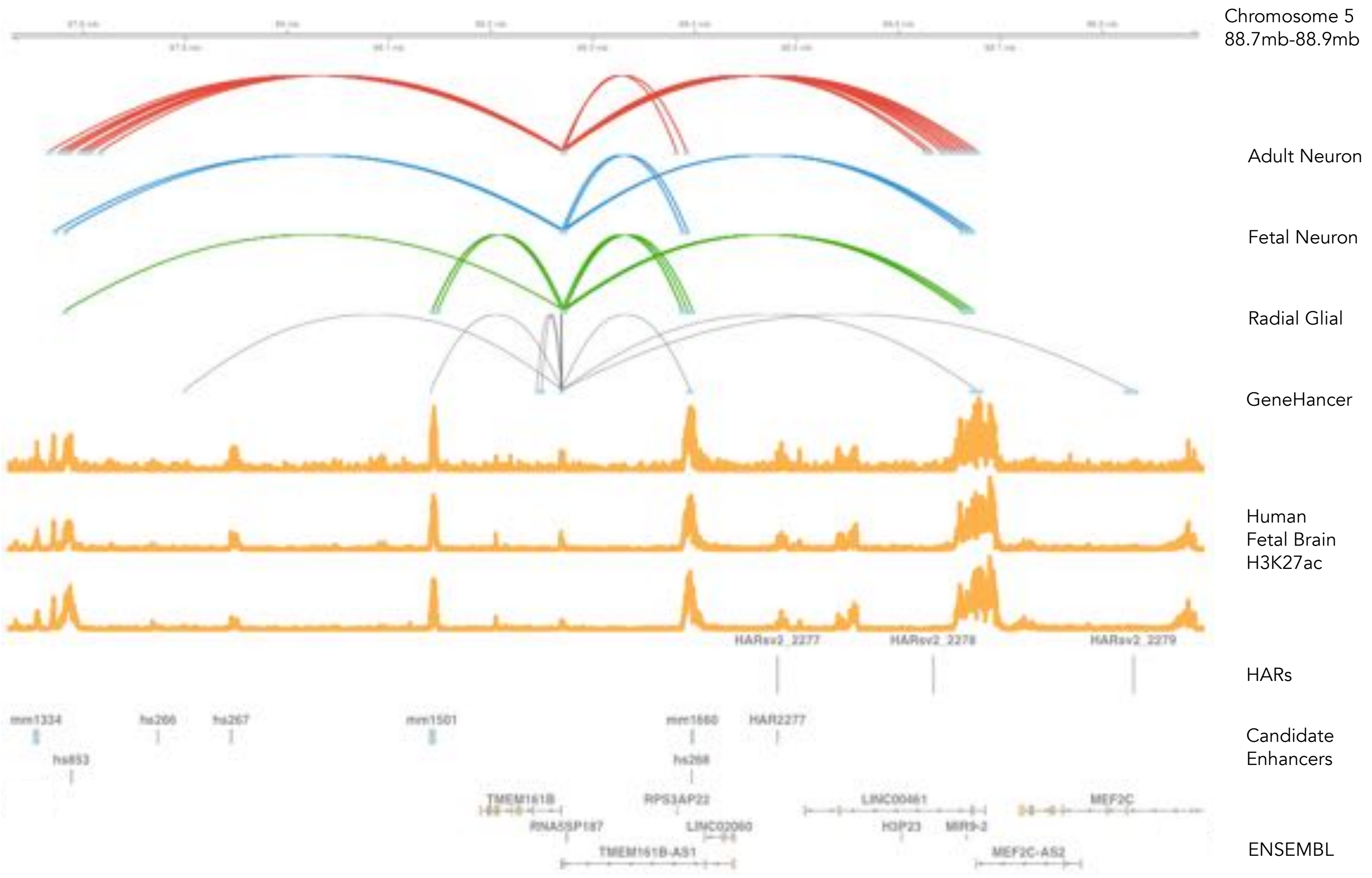

B **Bulk RNA seq in human cortex demonstrating higher fetal expression of TMEM161B and TMEM161B-AS1 compared to adult**

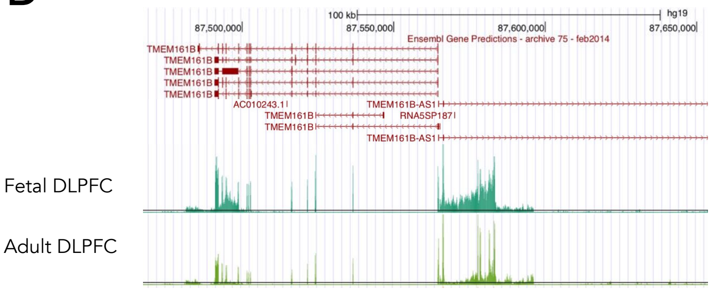

C **Generation of TMEM161B KO mouse from TMEM161B-LacZ reporter mouse**

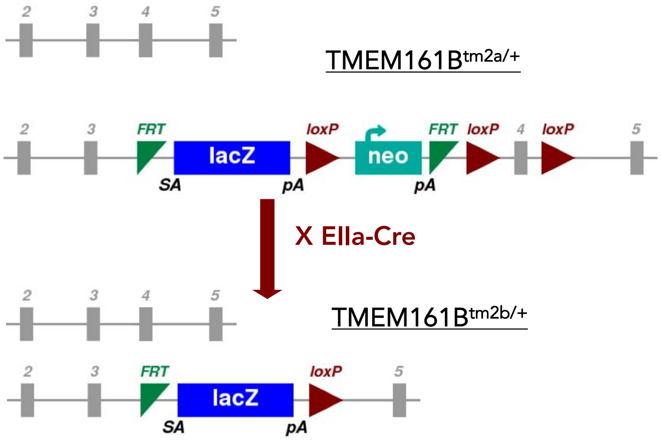

Figure S2: ***TMEM161B*** **regulation by local enhancers, expression in bulk-RNAseq, and generation of null-mouse line.**

(A) PLAC-seq data demonstrating interactions between local enhancers and the *TMEM161B* transcription start site. Enhancers sites described in Figure 2A show physical interaction with the TSS of *TMEM161B* in human radial glial or fetal neuron cells sequenced, confirming the enhancer-gene interaction in the developing CNS.

(B) Expression data of *TMEM161B*, *TMEM161B-AS1* in bulk RNAseq of human brain. *TMEM161B* is more highly expressed in the developing frontal cortex than it is in the mature brain, data analyzed from Jaffe et al. 2015, Nature Neuroscience.

(C) Breeding strategy for generating *Tmem161b* KO animals. *Tmem161b* null mice were generated through excision of exon 4 of *Tmem161b* via use of EIIa-cre breeder against the *Tmem161b*$^{tm2a/+}$ line described in STAR methods. This line also allows the reporting of *Tmem161b* transcriptional activity through b-galactosidase staining as demonstrated in Figure 2C. Null mice were confirmed via bulk-RNA sequencing as seen in Figure S3A and Figure S2D.

Expression of *TMEM161B* in human fetal brain

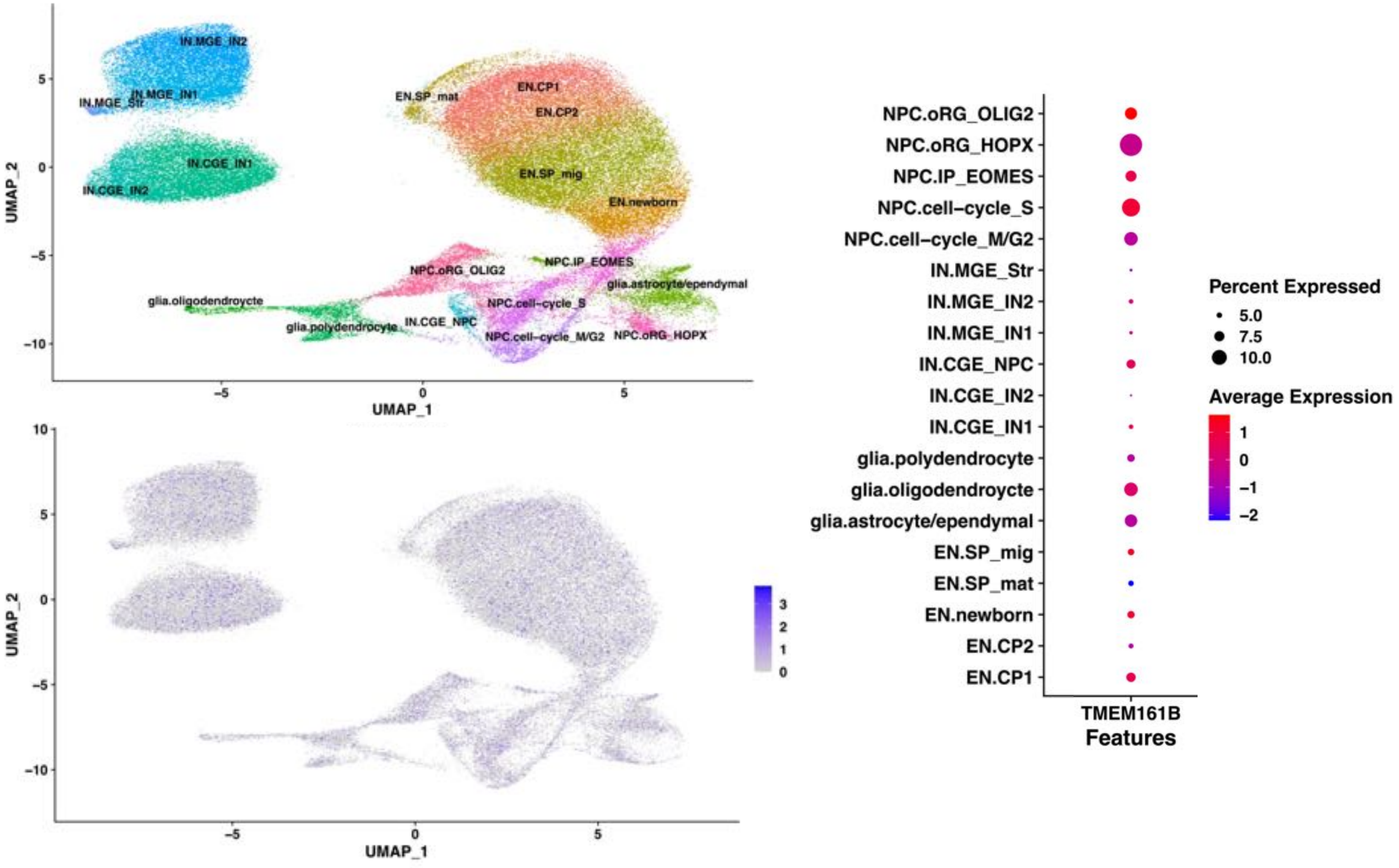

Figure S3: ScRNAseq expression of *TMEM161B* in human fetal cortex.

UMAP plot of single cell RNA expression of *TMEM161B* in human fetal brain.[28] Single cell RNA sequencing of human fetal cortex at gestational week 17, *TMEM161B* is diffusely expressed across cell types. Dot plot of cell-type RNA expression of *TMEM161B* in human fetal brain summarizes expression across cell types. *TMEM161B* shows more expression in progenitor cell types relative to mature ones and is present in outer radial glial cells implicated in cortical folding such as HOPX+ oRGs. NPC = neural progenitor cell, oRG = outer radial glial cell, IP = intermediate progenitor, IN = inhibitory neuron, MGE = medial ganglionic eminence, CGE = caudal ganglionic eminence, EN = excitatory neuron, SP = subplate, CP = cortical plate.

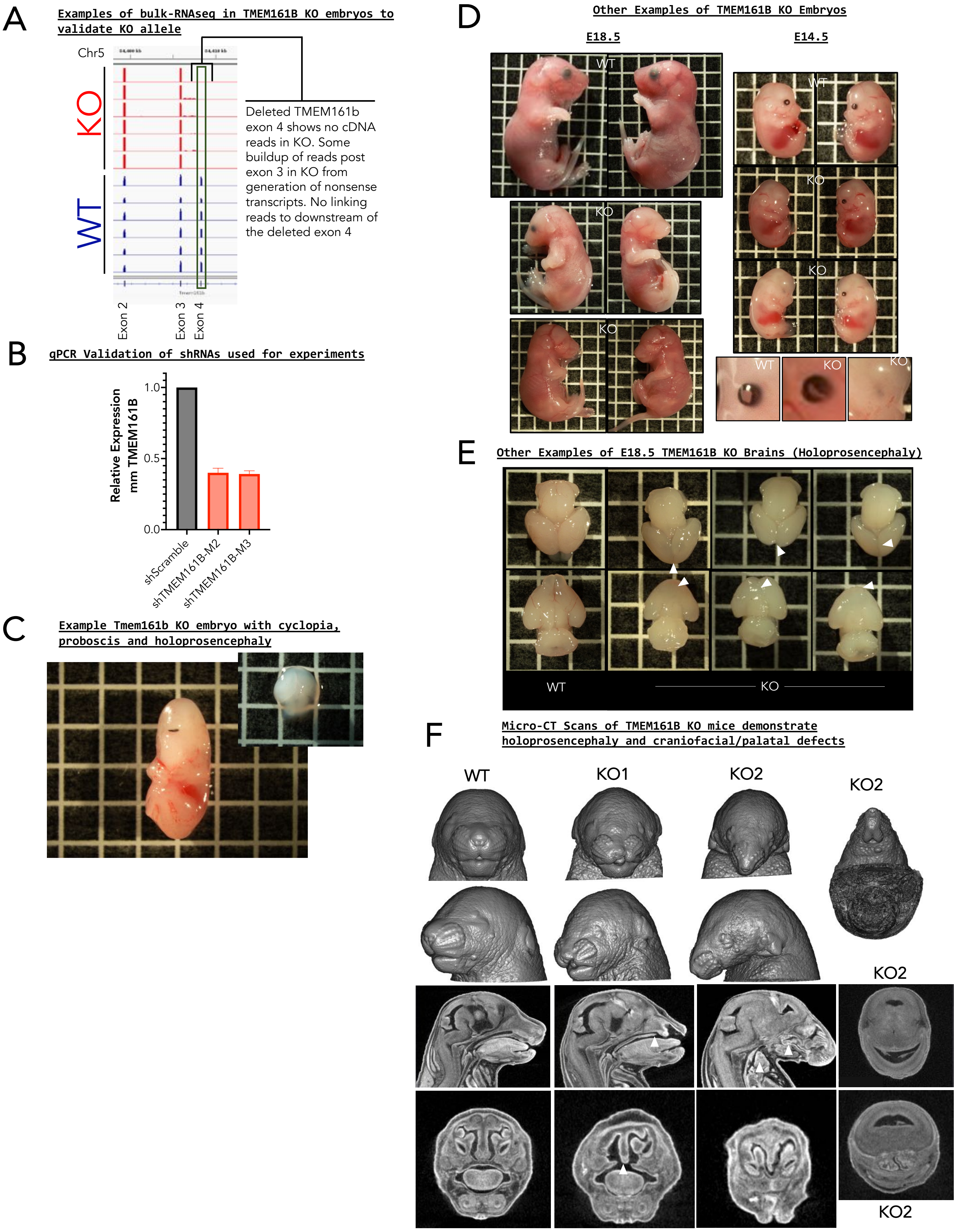
A
Examples of bulk-RNAseq in TMEM161B KO embryos to validate KO allele
Chr5
KO
WT
Deleted TMEM161b exon 4 shows no cDNA reads in KO. Some buildup of reads post exon 3 in KO from generation of nonsense transcripts. No linking reads to downstream of the deleted exon 4
Exon 2
Exon 3
Exon 4
B
qPCR Validation of shRNAs used for experiments
Relative Expression mm TMEM161B
1.0
0.5
0.0
shScramble
shTMEM161B-M2
shTMEM161B-M3
C
Example Tmem161b KO embryo with cyclopia, proboscis and holoprosencephaly
D
Other Examples of TMEM161B KO Embryos
E18.5
E14.5
WT
KO
KO
WT
KO
KO
WT
KO
KO
E
Other Examples of E18.5 TMEM161B KO Brains (Holoprosencephaly)
WT
KO
F
Micro-CT Scans of TMEM161B KO mice demonstrate holoprosencephaly and craniofacial/palatal defects
WT
KO1
KO2
KO2
KO2
KO2

Figure S4: Further characterization of *Tmem161b* null mouse

(A) Validation of the *Tmem161b* KO allele. Comparing bulk-RNAseq of E14.5 forebrain from *Tmem161b* WT and null embryos demonstrated that the null allele functioned as designed, producing transcript that has no exon 4 reads or linking reads to any downstream exons.

(B) qPCR Validation of shRNA-M2 and shRNA-M2 targeting ***Tmem161b*.** Scramble shRNA, shRNA Tmem161b-M2 (used in *in utero* and SL2 assays), and shRNA Tmem161b-M3 (used in SL2 assays) plasmids were transfected onto the N2A or SL2 cultures with Lipofectamine 3000 reagent (Thermo Fisher) according to manufacturer protocol. The cells were checked under fluorescence for GFP reporter expression to estimate transfection efficiency at 48 hours. The transfection efficiency was approximately 50-60% when the cells were harvested after 96 hours of incubation, using a Qiagen RNEasy RNA extraction kit. mRNA was isolated from the RNA extracts through SuperScript VILO cDNA synthesis using poly-A priming. qPCR was performed on diluted cDNA using PowerUP SYBR Green Master Mix with 5 technical replicates in qPCR to estimate the amount of TMEM161B transcript in the Scramble vs. TMEM161B shRNA conditions. Both shRNAs demonstrated excellent knockdown of >50% in this experiment that included both transfected and untransfected cells.

(C-E) Additional examples of *Tmem161b* KO embryo gross anatomy. C: An example of a particularly severely affected *Tmem161B* KO embryo that showed frank cyclopia, proboscis, and holoprosencephaly. D: Examples of the range of gross phenotypes observed in *Tmem161b* KO embryos at age E18.5 and E14.5 demonstrating eye and craniofacial defects as described in the main text. E: Examples of holoprosencephaly in *Tmem161b* KO embryos at E18.5.

(F) Micro-CT scans of *Tmem161b* KO embryos. Micro CT-scans of two *Tmem161b* KO embryos demonstrate (KO1) mild cleft palate with incomplete palatal fusion, or (KO2) complete failure of craniofacial development leading to proboscis-like structure in a severely affected KO embryo.

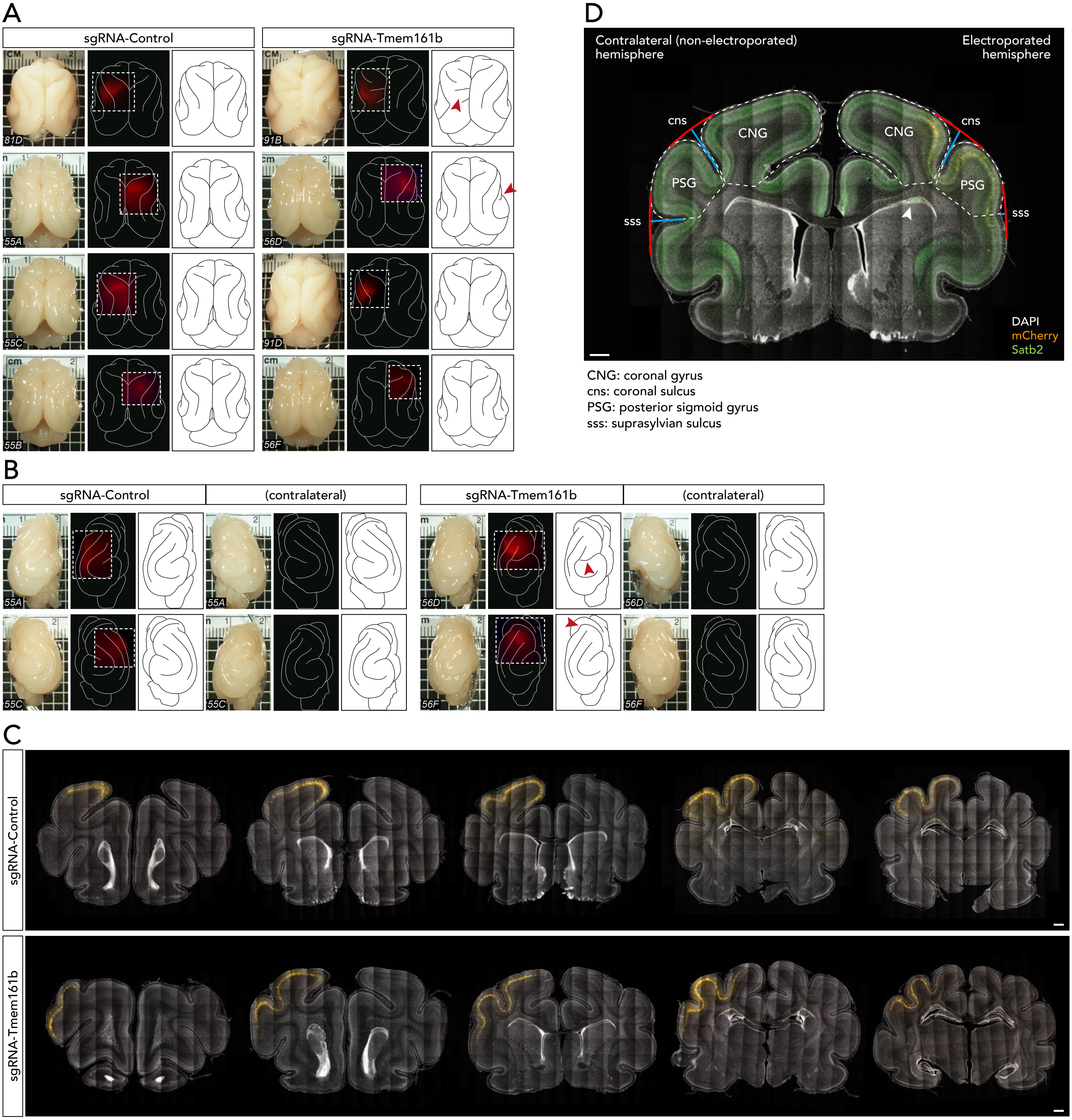

A
sgRNA-Control
sgRNA-Tmem161b
81D
55A
55C
55B
91B
56D
91D
56F
B
sgRNA-Control
(contralateral)
sgRNA-Tmem161b
(contralateral)
55A
55C
56D
56F
C
sgRNA-Control
sgRNA-Tmem161b
D
Contralateral (non-electroporated) hemisphere
Electroporated hemisphere
cns
CNG
PSG
sss
DAPI
mCherry
Satb2
CNG: coronal gyrus
cns: coronal sulcus
PSG: posterior sigmoid gyrus
sss: suprasylvian sulcus

Figure S5: *In vivo* CRISPR/Cas9-mediated knockdown of *Tmem161b* in the developing ferret cortex.

(A) Views of P21 ferret brains electroporated with plasmid encoding single-guide RNA (sgRNA) against *Tmem161b* or control plasmid. Squared boxes with fluorescent images show mCherry expression indicating the electroporated region. Red arrows indicate the gross anatomical abnormalities in gyri and sulci within the electroporated regions of *Tmem161b*-knockdown ferret brains.

(B) Lateral views of electroporated brains showing the electroporated target region. Note the absence of fluorescent signal in the contralateral hemispheres, which is not targeted by the *in utero* electroporation.

(C) Additional examples of tile scan images of coronal ferret brain sections showing the electroporated cortical areas of a control and *Tmem161b*-knockdown brain.

(D) Coronal section of *Tmem161b*-knockdown brain showing the somatosensory cortex with electroporated mCherry+ neurons. Coronal section immunostained with Satb2 as a marker of collosal neurons (green) and counterstained with DAPI (white). White arrow highlights the presence of groups of Satb2+ neurons intermingled with mCherry+/Satb2+ neurons that failed to reach the cortical column, which was consistently observed in all four *Tmem161b*-knockdown ferret brains. Blue lines indicate the sulcal depth measurements of the suprasylvian sulcus and the coronal sulcus. Dotted white lines indicate the gyrus size measurements of the coronal gyrus and the posterior sigmoid gyrus. These anatomical variables were measured in both the electroporated and non-electroporated hemispheres to estimate the local ratio of gyrus size and sulcal depth quantified in Figure 4. Scale bars, 1 mm (C-D).

E13.5->P7 In Utero Electroporation of Tmem161b shRNA (GFP) – co-stained with CTIP2 and SATB2

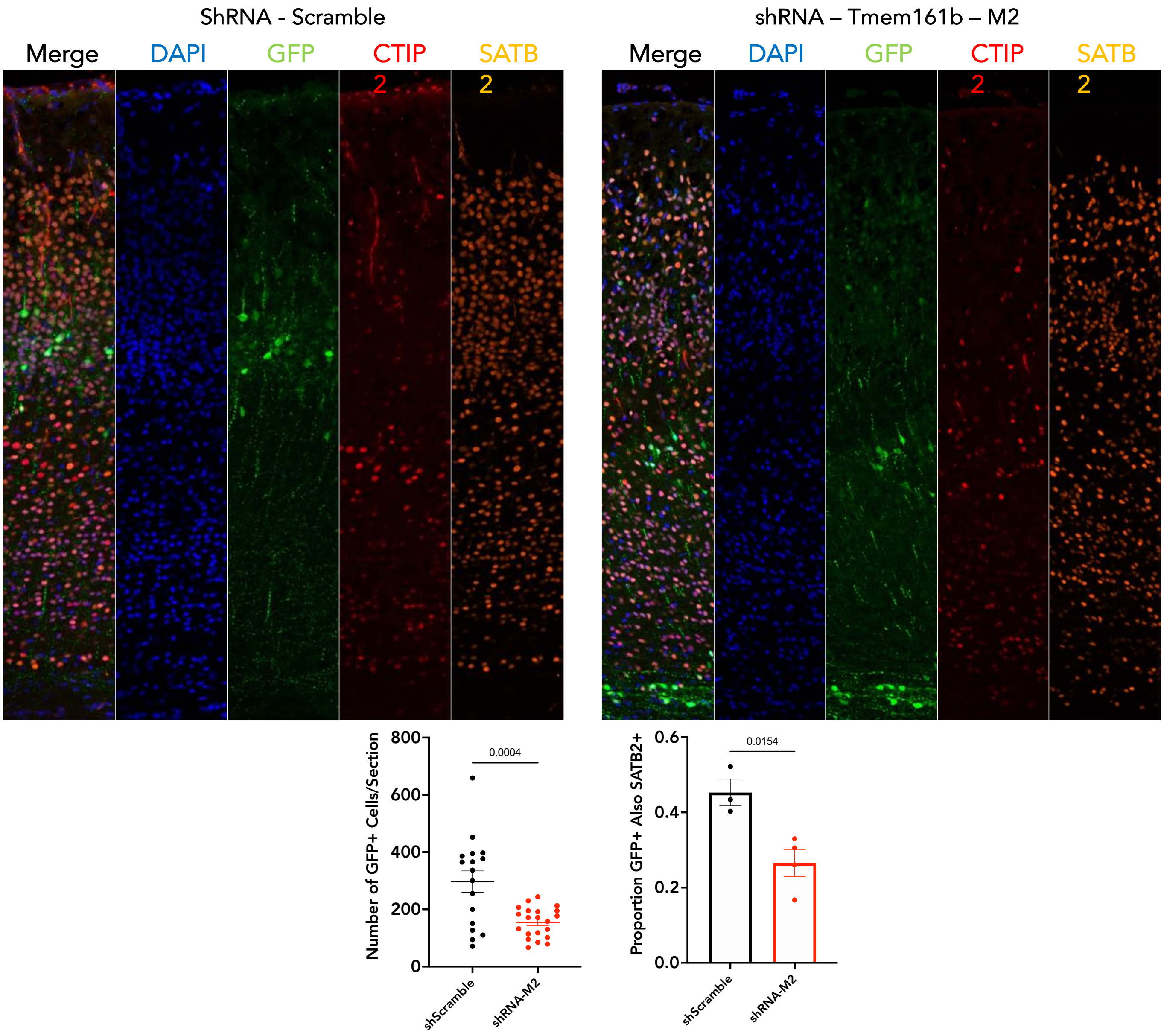

Figure S6: Related to Figure 4C-D

IUE performed at E14.5, and analysis at P7, where final distribution of GFP+ cells was quantified across 10 laminar bins of the P7 cortex divided evenly from pial surface to bottom of cortex was described in Figure 4. Knockdown of *Tmem161b* at E14.5 led to an altered distribution of GFP+ cells at P7 (2-way ANOVA of plasmid condition X cortical region showed an interaction effect, $p<0.001$, with post Sidak multiple comparison tests, corrected for multiple testing). Co-staining these same slides with Satb2 and Ctip2 as layer markers revealed a negligible amount of GFP+/Ctip2+ co-stained cells, but a depletion of GFP+/Satb2+ double marked cells in the *Tmem161b* knock-down condition relative to controls (t-test, n=3 control, n=4 knockdown, $p=0.0154$). Additionally, there were fewer overall GFP+ cells in the knockdown condition relative to the control condition, suggesting that disruption of *Tmem161b* led to fewer overall progeny (t-test, $p=0.0004$).

A

Quantification of Arl13b+ puncta in Tmem161b KO apical cortex IF

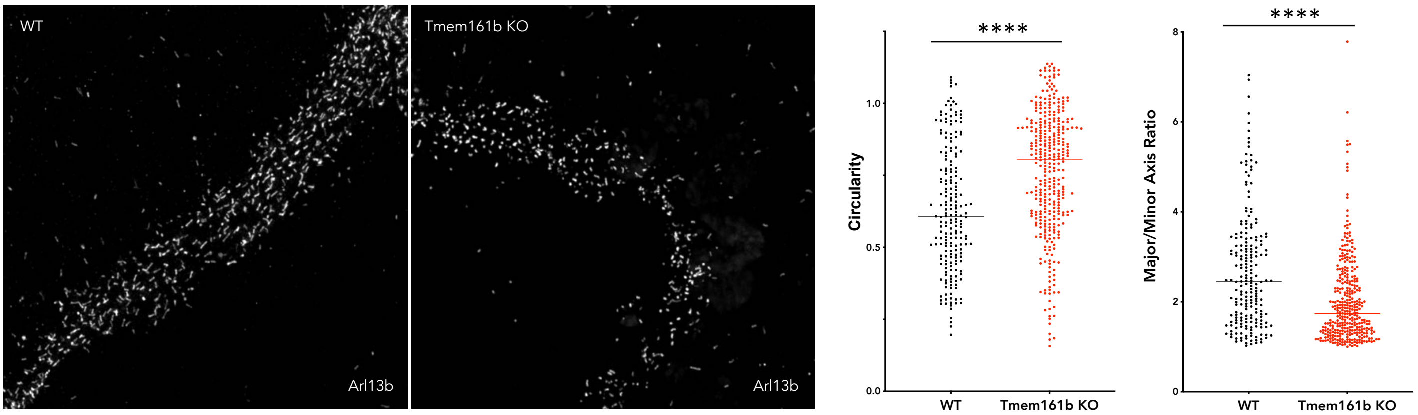

B

Additional SEM Images of Tmem161b KO Apical Cortex

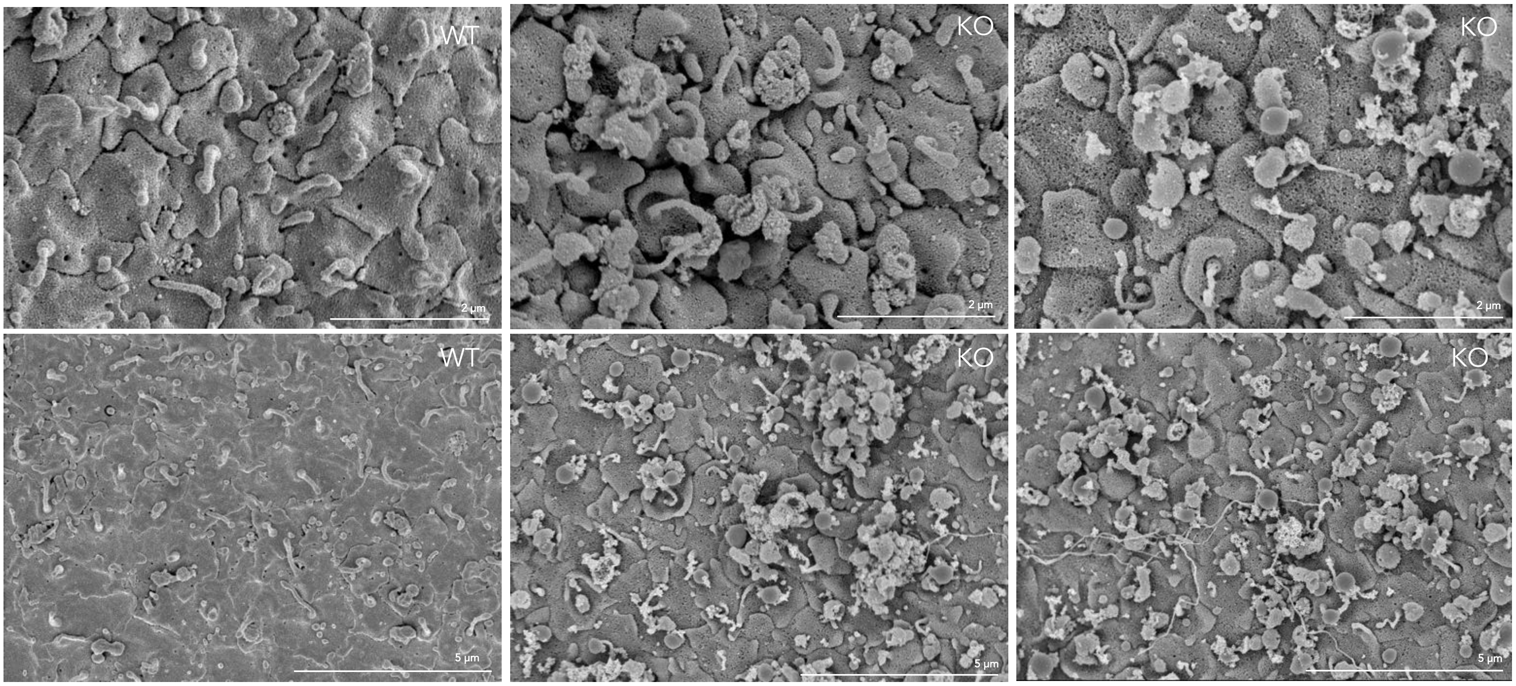

Figure S7: Related to Figure 5

(A) Additional examples of SEM of the *Tmem161b* KO apical ventricular surface at E14.5. WT ventricular surfaces show multiple healthy-looking cilia ranging from 1-2 um in visible length with minimal vesicular debris present on the apical membrane surface. KO samples show dysmorphic cilia with ciliary tip ballooning and vesicular debris as described in the main text.

(B) Example of Image Quantification of IF of Arl13b+ puncta at ventricular zone of E14.5 mouse embryos. Although *Tmem161b* WT and KO mice show the same number of Arl13b+ puncta on IF, these puncta are different in size/shape – analysis of the images under super-resolution confocal microscopy demonstrate that *Tmem161b* KO cilia show increased circularity (suggesting shorter length), and a decreased major/minor axis ratio suggesting the same. These differences are better appreciated on electron microscopy as above.

Supplementary Video 1 – Related to Figure 5E
XZ Plane video of inset region showed in Figure 5E. Green signal corresponds to Tmem161b-citrine, and red signal to Acetylated tubulin. Note the green signal as puncta at multiple ciliary tips as well as ciliary bases.

Supplementary Video 2 – Related to Figure 5F
Video of z-stack from maximum intensity projection displayed in Figure 5F of ciliated IMCD3 cells with transfected humanized TMEM161B-GFP, co-stained for Acetylated tubulin. Green signal corresponds to Tmem161B-GFP fusion protein, and red signal to Acetylated tubulin. Note accumulation of GFP+ signal at ciliary tips in this field.

Clinical Case Summaries of Individuals with biallelic *TMEM161B* variants

Family A
Individual: 09DG00538

09DG00538 was born to a consanguineous Caucasian couple at term by spontaneous vaginal delivery following an uneventful pregnancy. Birth weight was 3.5 kg (0.2 SD), she was noted to have congenital microcephaly, and seizures commenced within 1 hour after birth necessitating neonatal intensive care. Brain MRI revealed extensive bilateral polymicrogyria and ventriculomegaly, with some heterotopic subependymal grey matter nodules. Her epilepsy was never controlled despite treatment with many antiepileptic drugs. Examination at 3.5 years old showed axial hypotonia with spastic quadriplegia and, apart from severe microcephaly and related craniofacial changes, there was no gross dysmorphism. The family history is notable for a brother with microcephaly who died at 4.5 years of age but no other records are available. Exome sequencing revealed an inherited, homozygous missense variant in *TMEM161B* [NM_153354.5: c.1139C>T; p.(Ala380Val)]. Both parents and an unaffected sibling are carriers of this variant; no sample from the predeceased affected brother was available.

NM_153354.4(TMEM161B):c.1139C>T; p.(Ala380Val)
Chr5(GRCh37): g.87493533G>A
CADD: 23.0
MutationTaster (v2021): Deleterious. Tree vote: 64|36 (del|benign)
SIFT (v6.2.0): DELETERIOUS (score: 0.02, median: 3.34)
PolyPhen2: BENIGN (score: 0.310)
Highly conserved nucleotide (phyloP: 8.16 [-19.0, 11.0])
Highly conserved amino acid

<u>Family B</u>
Individual: UDN172478

UDN172478 is a 4-year-old male. He was born at term by induced vaginal delivery to a nonconsanguineous Caucasian couple. The pregnancy was complicated by mild preeclampsia around 35 weeks and oligohydramnios at 38 weeks. He was evaluated in the first year of life for developmental delay and was noted to have central hypotonia with bilateral upper and lower extremity hypertonia (left more severe than right), consistent mild spastic quadriparesis. Around 11 months of age he was evaluated for seizures due to head drop and spasm-like movements, but an EEG was normal, even during the head drop and jerking episodes. Brain MRI revealed extensive bilateral polymicrogyria most severe in the Perisylvian cortex. Developmental assessment at 3 years of age confirmed global developmental delay. His gross motor skills were at a 12-month level [Developmental quotient (DQ): 33%, severe delay]; visual-motor/problem solving was at a 17.2-month level (DQ: 48%, moderate delay); and speech/language was at an 11.8-month level (DQ: 33%, severe delay). His social-emotional skills were considered appropriate for his developmental age at that time. At 3.5 years, he was nonverbal but was able to use about 10 signs. His receptive language skills are better than his expressive language. Prior testing included negative IgG for CMV, normal auditory brainstem responses, and a chromosomal microarray that identified an 8q21.3 duplication of about 1.3 Mb that contains no known genes (chr8:89431761-90702451, hg19). Exome sequencing revealed compound heterozygous *TMEM161B* variants, a missense variant [NM_153354.5: c.980T>C; p.(Leu327Ser) paternal] and an intronic variant [chr5:87501626C>T (hg19), c.800+5G>A maternal] predicted to impact splicing (Figure S1, D).

NM_153354.4(TMEM161B):c.980T>C; p.(Leu327Ser)
Chr5(GRCh37): g.87494902A>G
CADD: 28.4
MutationTaster (v2021): Deleterious. Tree vote: 58|42 (del|benign)
SIFT (v6.2.0): DELETERIOUS (score: 0.01, median: 3.34)
PolyPhen-2: PROBABLY DAMAGING (score: 0.999)
Highly conserved nucleotide (phyloP: 9.33 [-19.0, 11.0])
Highly conserved amino acid

NC_000005.9(NM_153354.4):c.800+5G>A
Chr5(GRCh37):g.87501626C>T
CADD: 24.6
Predicted change at donor site 5 bps upstream: -71.6%
MaxEnt: -100.0%
NNSPLICE: -98.0%
SSF: -16.7%
Moderately conserved nucleotide (phyloP: 5.79 [-19.0, 11.0])

Family C
Individuals: BFP903, BFP904, BFP905 and BFP906

BFP906 was the eldest of four siblings born to unrelated Caucasian parents. All four siblings were reported to have chronic seizures and spastic quadriparesis with intellectual disability. BFP906 died suddenly at the age of 32 years. On autopsy, he was noted to have right ventricular cardiomyopathy with mild hypertrophy and dilatation, as well as increased adiposity. He was further noted to have macroglossia, scoliosis (despite Harrington rods), and to have been cachexic at time of death. On pathological examination of the brain, there was extensive bilateral, symmetric polymicrogyria affecting all regions of the cortex that was more severe in the Perisylvian region, as well as hypoplasia of the cerebral peduncles. The basal ganglia were normal in appearance, no heterotopias were noted, and the ventricles were mildly enlarged. The corpus callosum was present and of apparent normal thickness. The choroid plexus was normal appearing throughout the brain. Several small iron deposits, possibly consistent with old, small hemorrhages were noted (one dorsal to the left caudate nucleus, one ventral to the left caudate nucleus, and one dorsal to the right caudate nucleus). Microscopic examination of the cortex (see below) identified a reduced cortical thickness, multiple chaotic folds and many areas where the marginal zone extended through poorly formed clefts in the cortical surface. No cortical regions were identified to have a normal 6-layered cortex, there was noted radial disorganization of cell bodies, and the brain was depleted of granule cells. Although the three siblings of BFP906 (BFP903, BFP904 and BFP905) were all reported to have a similar neurological condition, no imaging was available on these individuals, and they were placed into full-time care under state proxy later in adulthood. Exome sequencing on three siblings revealed compound heterozygous variants in *TMEM161B* [NM_153354.5: c.580G>A; p.(Glu194Lys) paternal and c.362C>T; p.(Thr121Ile) maternal], their only shared candidate disease gene. Multiple attempts to sequence fixed tissue from BFP906 failed due to degraded DNA.

NM_153354.4(TMEM161B):c.362C>T; p.(Thr121Ile)
Chr5(GRCh37):g.87516464G>A
CADD: 24.6
MutationTaster (v2021): Deleterious. Tree vote: 73|27 (del|benign)
SIFT (v6.2.0): DELETERIOUS (score: 0.01, median: 3.34)
PolyPhen-2: PROBABLY DAMAGING (score: 0.999)
Highly conserved nucleotide (phyloP: 9.54 [-19.0, 11.0])
Moderately conserved amino acid

NM_153354.4(TMEM161B):c.580G>A; p.(Glu194Lys)
gDNA Level: Chr5(GRCh37):g.87502864C>T
CADD: 24.8
MutationTaster (v2021): Deleterious. Tree vote: 52|48 (del|benign)
SIFT (v6.2.0): DELETERIOUS (score: 0.03, median: 3.34)
PolyPhen-2: BENIGN (score: 0.018)
Highly conserved nucleotide (phyloP: 7.84 [-19.0, 11.0])
Highly conserved amino acid

Supplementary Discussion

Potential cellular roles for TMEM161B

The ciliary phenotype noted on SEM was dramatic but apparently not catastrophic in its effect; the phenotype of the *Tmem161b* KO mouse is not as severe as those that obliterate function of the primary cilium entirely[1,2], and only a proportion of cilia observed at the moment of fixation exhibited ballooning in our KO embryos, estimated at 10-80% depending on the field observed. Thus, one hypothesis of how pathogenic variants in *TMEM161B* lead to the ciliary abnormalities is that *TMEM161B* may play a role in the proteostasis of transmembrane receptors that are normally regulated through exquisitely precise ciliary trafficking. The SEM of abnormal primary cilia in *Tmem161b* KO mice is reminiscent of, though more dramatic than, abnormal cilia found in mutants of ciliary trafficking proteins, such as *Ift140*[3], *Ift144*[4], *Dync2h1*[4], or the Golgi protein *Golgb1*[5], all phenotypes of which are due to material accumulating in the ciliary tip.

Another possibility is that Tmem161b may be involved in the machinery that regulates actin-dependent ciliary vesicle exocytosis, defects in which can lead to abnormal Shh signaling, while not impairing an initial Smo translocation into the primary cilium.[6] Receptors and other transmembrane proteins that do not undergo retrograde transport in the cilium can be released through ciliary exocytosis, and poisoning actin remodeling in ciliated cells responding to ciliary GPCR ligands with cytochalasin D causes accumulation of receptors in the ciliary tip with consequent ballooning.[6] Early ciliary disassembly of the ciliary tip is based on similar mechanisms[7], so the ciliary abnormalities seen in our *Tmem161b* null embryos could be related to a failure of scission of either ciliary exocytic vesicle release or ciliary decapitation. These hypotheses (trafficking defect, exocytic vesicle release defect, and ciliary tip release defect) are not mutually exclusive, and future studies using live cell imaging might help describe the changes in ciliary dynamics in *TMEM161B* null cells and help illuminate the specific cellular function of TMEM161B.

Supplementary Methods

Airyscan Super-Resolution Confocal Microscopy
Coronal cryosections of E14.5 and E18.5 mouse cortex were immunostained for primary cilia marker *Arl13b* and imaged using a Zeiss LSM 980 confocal microscope with Airyscan 2. Imagining was performed under "Super Resolution" mode of Airyscan at 63X magnification. Images were captured at the apical surface of the dorsolateral corner of the lateral ventricles to maintain consistency of the anatomical locations. The Z-stack function was used to image a 5 µm thickness, and automatic Airyscan processing was performed after acquisition. All images were captured at the same imaging settings of laser intensity and detector gain. Sample images were prepared in ImageJ and Zen software.

Primary Cilia Quantification
Image analysis was performed automatically using ImageJ software. Maximal intensity projection and automated thresholding was performed on the image. A mask of all the cilia was generated on the thresholded image using the Analyze Particles function in ImageJ. To exclude noise pixels and overlapping cilia, a size range of 0.05 – 3 µm2 was selected. ImageJ automatically determines an ellipse fit for each cilium and the output contains the area and the perimeter of the cilium, and the major axis length and minor axis length of the fitted ellipse. The circularity was calculated as "4*π *area/ perimeter2", a value closer to 1 indicates more rounded shape. The major/minor ratio is calculated by the ratio between the length of the major and minor axis of the fitted ellipse, a larger value indicates more elongated cilium.

Analysis of scRNA-seq data
To facilitate cross-species comparison of TMEM161b expression, an integrated fetal brain scRNA-seq atlas was created from the following publicly available datasets:

1) Polioudakis et al., Neuron 2019 (http://solo.bmap.ucla.edu/) [Human]
2) Fan et al., Cell Res. 2018 (GEO: GSE103723) [Human]
3) Nowakowski et al., Science 2017 (https://cells.ucsc.edu/?ds=cortex-dev) [Human]
4) Zhong et al., Nature 2018 (GEO: GSE104276) [Human]
5) Smith et al., PNAS 2021 (dbGAP: phs001272.v1.p1) [Human]
6) Johnson et al., Nature 2018 (GEO: GSE110010) [Ferret]
7) Ruan et al., PNAS 2021) (GEO: GSE161690) [Mouse]

The dataset from Polioudakis et al., showed strong clustering by tissue donor, and, as recommended by Seurat developers, was first integrated across donors using the Seurat integration pipeline. This integrated dataset as well as all other datasets above was then processed individually using a consistent quality-control pipeline, including filtering of features expressed in <3 cells, cells that express an unusually high or low number of features, and cells with >5% mitochondrial transcripts. Seurat's *scTransform* pipeline was used for normalization, variance stabilization and regression of confounding sources of variation, such as batch, percent mitochondrial expression, difference between cell-cycle phases, and percent ribosomal expression.

After processing each dataset individually, all datasets were integrated together using Seurat's standard integration pipeline to create the final integrated fetal brain scRNA-seq atlas. Dimensionality reduction was performed to visualize the atlas, and visualization of covariates such as species and dataset of origin showed that clustering was not being driven by origin effects.

Unsupervised clustering was performed using the standard Seurat pipeline, and cell types were annotated based on a comprehensive, multi-person approach utilizing both marker gene expression and analysis of each cell's originally assigned cell type identity within its dataset of origin. TMEM161b's expression profile was visualized across these final cell type identities.

Human Genetics Sequencing

DNA extracted from peripheral blood of enrolled subjects was analyzed by exome sequencing (ES). ES and data processing for Family A was performed by the Genomics Platform at the Broad Institute of Harvard and MIT (Cambridge, MA, USA). ES was performed on DNA samples (>250 ng of DNA, at >2 ng/μl) using Illumina exome capture (38 Mb target). The ES pipeline included sample plating, library preparation (2-plexing of samples per hybridization), hybrid capture, sequencing (150 bp paired reads), sample identification quality control check, and data storage. The hybrid selection libraries cover >90% of targets at 20x and a mean target coverage of ~100x. The ES data was de-multiplexed and each sample's sequence data were aggregated into a single Picard BAM file. ES data were processed through a pipeline based on Picard, using base quality score recalibration and local realignment at known indels. The BWA aligner was used for mapping reads to the human genome build 37 (hg19). Single nucleotide polymorphism (SNPs) and insertions/deletions (indels) were jointly called across all samples using Genome Analysis Toolkit (GATK) HaplotypeCaller package version 3.4. Default filters were applied to SNP and indel calls using the GATK Variant Quality Score Recalibration (VQSR) approach. Lastly, the variants were annotated using Variant Effect Predictor (VEP). For additional information please refer to Supplementary Section 1 of the paper describing ExAC.[8] The variant call set was uploaded on to Seqr and analysis was performed to identify candidate variants according to ACMG guidelines. ES was performed for Family B and Family C via similar methods.

Candidate variants identified via ES were evaluated by manual review for their rarity, predicted effects, and review of literature. For each family, following the emergence of *TMEM161B* as the most likely candidate gene, confirmation of the variants by Sanger sequencing was performed on original subject samples in each family, including first degree relatives, when available, to confirm segregation of *TMEM161B* alleles with phenotype. In silico prediction of the effect of missense variants was performed using Combined Annotation Dependent Depletion (CADD; https://cadd.gs.washington.edu), MutationTaster (www.mutationtaster.org), SIFT (https://sift.bii.a-star.edu.sg), and PolyPhen-2 (http://genetics.bwh.harvard.edu/pph2). The splice region variant was characterized using Alamut® Visual Plus v1.3 (Sophia Genetics) by MaxEntScan, NNSPLICE, and SpliceSiteFinder-like.

Micro CT scans

Embryos were dissected into ice-cold phosphate buffered saline (PBS) and exsanguinated by severing the umbilical vessels. After washing in PBS, embryos were fixed by immersion in 4% paraformaldehyde (PFA in PBS) for 7 days for embryos aged E18.5 (or 24 hours for E14.5) at 4°C, before storing in 1% PFA at 4°C. Embryos were contrasted by immersion in 50% Lugol's solution for 2 weeks, protected from light, and solution exchanged for fresh every 2 days (for 2 days total for E14.5s). After contrasting, embryos were washed in ddH_2O for at least 1 hour before embedding in an acrylic mount in 1% agarose dissolved in ddH_2O and allowed to set for at least 2 hours. Micro-CT data sets were acquired using a Skyscan 1172 scanner (Bruker). With x-ray source set to 80kV and using 0.5-mm aluminum filter, two averaged projections were acquired every 0.25° through a total rotation of 180° at 5μm/pixel resolution (3μm for E14.5s). NRecon (Bruker) was using for 3D

reconstruction, followed by cropping and scaling to 28μm (14μm for E14.5s) isotropic voxels using the HARP software.[9]

Antibodies Utilized
Primary antibodies utilized in IF experiments included: rabbit anti-DsRed, 1:500 (Clontech, Cat #632496); chicken anti-GFP 1:300-500 (Abcam, ab13970); rabbit anti-Pax6 1:500 (EMD Milipore, ab2237); mouse anti-Pax7 1:20 (DSHB, Pax7); rabbit anti-Arl13b 1:1000 (Proteintech, 17710-1-AP); mouse anti-Acetylated tubulin 1:1000 (Sigma-Aldrich, T6793); rabbit anti-FoxA2 1:500 (Cell Signaling, 8186S); mouse anti-Shh 1:10 (DSHB, 5E1); mouse anti-Nkx2.2 1:100 (DSHB, 745A5); mouse anti-Nkx6.1 1:100 (DSHB, F55A10); rabbit anti-Olig2 1:250 (EMD Milipore, ab9610).

Molecular Cloning/shRNA Validation
shRNA plasmids were ordered from VectorBuilder, using their shRNA plasmid generation tool on a pRP background, to include shRNA sequences specific to mouse *Tmem161b*. The efficiency of these plasmids was evaluated in two different mouse cell lines before use in experiments. SL2 cells were grown as adherent cultures to 60% confluency. Scramble shRNA, shRNA Tmem161b-M2 (used in *in utero* and SL2 assays), and shRNA Tmem161b-M3 (used in SL2 assays) plasmids were transfected onto the N2A or SL2 cultures with Lipofectamine 3000 reagent (Thermo Fisher) according to manufacturer protocol. The cells were checked under fluorescence for GFP reporter expression to estimate transfection efficiency at 48 hours. The transfection efficiency was approximately 50-60% when the cells were harvested after 96 hours of incubation, using a Qiagen RNEasy RNA extraction kit. mRNA was isolated from the RNA extracts through SuperScript VILO cDNA synthesis using poly-A priming. qPCR was performed on diluted cDNA using PowerUP SYBR Green Master Mix with 5 technical replicates in qPCR to estimate the amount of TMEM161B transcript in the Scramble vs. TMEM161B shRNA conditions (Figure S4B).

Ferret Histology/Immunofluorescence
Ferret kits (postnatal day 21, P21) were deeply anesthetized with ketamine and xylazine by intraperitoneal injection, and transcardially perfused with PBS followed by 4% paraformaldehyde (PFA) in PBS. Dissected brains were post-fixed for 48 hr at 4°C, and then cryoprotected by immersion in a series of sucrose solutions (from 10% to 20% and 30% sucrose solutions in PBS, changed every 24 hr). Ferret brains were cut frozen on a sliding microtome (Leica SM2010 R) at 50 μm. Slices were mounted onto charged SuperFrost Plus slides (Fisher Scientific, Cat #22-037-246). Imaging of ferret brains was performed in a LSM980 confocal microscope at 8-bith depth with 5X/0.16 (Magnification/Numerical Aperture) objective and 1.0 digital zoom.

Tile scan images of coronal sections were acquired at 1024 x 1024 pixel resolution at 0.25 μs/pixel acquisition speed. Imaging of neuronal density and laminar position was performed at 2780 x 2780 pixel resolution at 0.68 μs/pixel acquisition speed, with 2 average per frame. Histological analyses were conducted at P21, an age in which neurogenesis and neuronal migration are largely completed in both somatosensory and motor regions (Smart and McSherry, 1986, Journal of Anatomy). For analysis of gross anatomical features, >10 coronal sections were examined for each ferret brain sample. Analysis of dorsal cortical surface was restricted to the somatosensory and motor cortices since the electroporated cells were predominantly found in these regions. Local gyrification was calculated as previously described (Matsumoto et al., 2020, eLife). Briefly, we calculated the ratio of the size of the gyri in the electroporated hemisphere compared to the size of the corresponding gyri in the contralateral, non-electroporated hemisphere; quantification of sulcal depth was also estimated as a ratio relative to the corresponding sulcus in the contralateral, non-electroporated hemisphere

(Fig. S5). We focused our analyses on the posterior sigmoid gyrus, the coronal gyrus, the coronal sulcus, and the suprasylvian sulcus, where the somatosensory and motor cortices lie, since electroporated cells were predominantly found in this region. Analysis of Satb2+/mCherry+ neurons across layers was restricted to the primary somatosensory cortex to consistently examine laminar organization across brain sections and reduce the variability inherent to regional differences.

IMCD3 Immunofluorescence

IMCD3 cells were fixed in 4% PFA for 10min and washed with PBS twice. The cells were then blocked (1% BSA in PBS) for 30min and incubated in primary antibodies (GFP 1:1000 (AbCam Cat# ab13970), Arl13b 1:300 (ProteinTech Cat# 17711-1-AP) and Acetylated Tubulin 1:500 (Sigma Cat# T6793) overnight at 4°C. Cells were then washed twice with PBS and incubated in secondary antibodies (Alexa Fluor 488/594/647 1:500) for 2 hours and mounted in Vectashield with DAPI (Vector Laboratories Cat #H1200). Immunofluorescent images were taken on a Zeiss LSM900 using Airyscan with either 40x water immersion or 63x oil immersion objectives. Images acquired were then processed using either Zen (Zeiss), IMARIS (Bitplane) and ImageJ/FIJI.

Mouse IF

Mouse embryos were harvested at ages between E11.5-E18.5 and were dissected in cold PBS to isolate tissues of interest (brains, or thorax for spinal cords). This tissue was drop fixed in 4% paraformaldehyde overnight (14-18 hours), and then rinsed in PBS several times before being placed overnight in 30% sucrose solution. Tissues were frozen in OCT and cryo-sectioned at 12-18μm. Slides were rinsed with PBS X5, and then blocked in a solution with 8% normal donkey serum + 0.3% BSA and 0.3% Triton X-100 for 45 minutes at room temperature before being treated with primary antibodies (see below) overnight at 4°C diluted in the blocking solution. Slides were rinsed with PBS X5 and then treated with secondary antibodies conjugated to Alexa fluorophores + DAPI diluted in blocking solution for 2 hours at room temperature before being rinsed, dried, and cover slipped with Prolong Diamond mounting medium. Slides were imaged on a (Zeiss LSM 550) confocal microscope.

Protein Modeling/Evolutionary Analyses

Remote homologs of human TMEM161b (https://www.uniprot.org/uniprot/Q8NDZ6) were harvested from sequence databases by iterative PSIBLAST[10] searches, and also aligned by the EVcouplings server (https://evcouplings.org).[11] More sensitive, proteome-level searches for distant TMEM161 family relatives were conducted by Hhpred at the MPI Bioinformatics toolkit (https://toolkit.tuebingen.mpg.de)[12] that also calculated PSIPRED secondary structure profiles[13] for the superfamily. The protein chains of TMEM161 family members typically displayed 9 transmembrane (TM) segments by Phobius analysis,[14] with the N-terminal TM1 showing a weak or equivocal signal peptide nature by SignalP5.[15] The three-dimensional structure of TMEM161b was first predicted by the top-ranking CASP14 servers trRosetta[16] and tFold,[17] convergently revealing a uniquely complex fold that did not bear any similarity to other membrane protein structures in the PDB[18] by DALI analysis.[19] A possible functional site in the TMEM161b fold was suggested by ConSurf analysis[20] that mapped an evolutionarily conserved pocket on the luminal face of the fold. The 3D structural models of TMEM161b and a phylogenetically broad range of homologs were greatly improved by ColabFold access (https://github.com/sokrypton/ColabFold)[21] to new AlphaFold2[22] and RosettaFold[23] deep learning-based programs, that employ end-to-end neural network algorithms with near-crystallographic accuracy.[24] This phylogenetically diverse ensemble of TMEM161b-related structures was used to locate conserved epitopes and residues in the unique fold, to help interpret mutations in human TMEM161b linked to disease and drive functional analysis. Structures manipulated and viewed with PyMOL (www.pymol.org).

Protein Modeling/Evolutionary Analyses

Remote homologs of human TMEM161b (https://www.uniprot.org/uniprot/Q8NDZ6) were harvested from sequence databases by iterative PSIBLAST[10] searches, and also aligned by the EVcouplings server (https://evcouplings.org).[11] More sensitive, proteome-level searches for distant TMEM161 family relatives were conducted by Hhpred at the MPI Bioinformatics toolkit (https://toolkit.tuebingen.mpg.de)[12] that also calculated PSIPRED secondary structure profiles[13] for the superfamily. The protein chains of TMEM161 family members typically displayed 9 transmembrane (TM) segments by Phobius analysis,[14] with the N-terminal TM1 showing a weak or equivocal signal peptide nature by SignalP5.[15] The three-dimensional structure of TMEM161b was first predicted by the top-ranking CASP14 servers trRosetta[16] and tFold,[17] convergently revealing a uniquely complex fold that did not bear any similarity to other membrane protein structures in the PDB[18] by DALI analysis.[19] A possible functional site in the TMEM161b fold was suggested by ConSurf analysis[20] that mapped an evolutionarily conserved pocket on the luminal face of the fold. The 3D structural models of TMEM161b and a phylogenetically broad range of homologs were greatly improved by ColabFold access (https://github.com/sokrypton/ColabFold)[21] to new AlphaFold2[22] and RosettaFold[23] deep learning-based programs, that employ end-to-end neural network algorithms with near-crystallographic accuracy.[24] This phylogenetically diverse ensemble of TMEM161b-related structures was used to locate conserved epitopes and residues in the unique fold, to help interpret mutations in human TMEM161b linked to disease and drive functional analysis. Structures manipulated and viewed with PyMOL (www.pymol.org).

Mouse Generation

The C57BL/6N-A^{tm1Brd}-$Tmem161b^{tm2a(EUCOMM)Hmgu}$/BayH mice (referred to as $Tmem161b^{tm2a}$) were originally generated by Baylor College of Medicine as part of their commitment to the International Mouse Phenotyping Consortium (IMPC), RRID: MMRRC_041541-UCD. These mice were bred against a pan-cre expressing line (e2a-Cre) to generate the $Tmem161b^{tm2b(EUCOMM)Hmgu}$ allele line (referred to as $Tmem161b^{tm2b}$). Initial phenotyping was conducted at The Mary Lyon Centre, MRC-Harwell. Some primary phenotyping data may be found at www.mousephenotype.org.

LacZ Staining E12.5

For LacZ staining all solutions were at pH 8.0. Embryos were dissected into ice-cold phosphate buffered saline (PBS) and exsanguinated by severing the umbilical vessels. After washing in PBS, embryos were fixed in ice-cold 4% paraformaldehyde (PFA) prepared in PBS for 20 minutes. For whole-mount staining, embryos passed through three, 30-minute PBS washes before being immersed in X-gal stain ($MgCl_2$ 2mM, IGEPAL CA-630 0.02%, potassium ferrocyanide 5mM, potassium ferricyanide 5mM, Sodium deoxycholate 0.01%, X-gal 1mg/mL, in PBS) and incubated at 4°C for 24 hours. Following staining embryos were rinsed and passed through two, 1-hour PBS washes, before post-fixing in 4% PFA overnight. For sections, after initial 20-minute fix, embryos were washed in PBS for 40 minutes, then transferred to 30% sucrose in PBS for cryoprotection overnight, frozen in OCT and stored at -80°C. 30µm sections were air dried, fixed in 4% PFA for 10 mins, passed through three, 5-minute PBS washes, before incubation in X-gal stain (as above, but with the addition of 12µg/mL 4-Nitro blue tetrazolium chloride) at 37°C for 48 hours.

Supplementary References

1. Liu, A., Wang, B. & Niswander, L. A. Mouse intraflagellar transport proteins regulate both the activator and repressor functions of Gli transcription factors. *Development* **132**, 3103–3111 (2005).
2. Huangfu, D. *et al.* Hedgehog signalling in the mouse requires intraflagellar transport proteins. *Nature* **426**, 83–87 (2003).
3. Miller, K. A. *et al.* Cauli: a mouse strain with an Ift140 mutation that results in a skeletal ciliopathy modelling Jeune syndrome. *PLoS Genet* **9**, e1003746 (2013).
4. Liem, K. F. *et al.* The IFT-A complex regulates Shh signaling through cilia structure and membrane protein trafficking. *J Cell Biol* **197**, 789–800 (2012).
5. Bergen, D. J. M., Stevenson, N. L., Skinner, R. E. H., Stephens, D. J. & Hammond, C. L. The Golgi matrix protein giantin is required for normal cilia function in zebrafish. *Biol Open* **6**, 1180–1189 (2017).
6. Nager, A. R. *et al.* An Actin Network Dispatches Ciliary GPCRs into Extracellular Vesicles to Modulate Signaling. *Cell* **168**, 252-263.e14 (2017).
7. Ikegami, K. & Ijaz, F. Current understandings of the relationship between extracellular vesicles and cilia. *The Journal of Biochemistry* **169**, 139–145 (2021).
8. Lek, M. *et al.* Analysis of protein-coding genetic variation in 60,706 humans. *Nature* **536**, 285–291 (2016).
9. Brown, J. M. *et al.* A bioimage informatics platform for high-throughput embryo phenotyping. *Brief Bioinform* **19**, 41–51 (2018).
10. Altschul, S. F. *et al.* Gapped BLAST and PSI-BLAST: a new generation of protein database search programs. *Nucleic Acids Res* **25**, 3389–3402 (1997).
11. Hopf, T. A. *et al.* Sequence co-evolution gives 3D contacts and structures of protein complexes. *Elife* **3**, (2014).
12. Zimmermann, L. *et al.* A Completely Reimplemented MPI Bioinformatics Toolkit with a New HHpred Server at its Core. *J Mol Biol* **430**, 2237–2243 (2018).
13. Buchan, D. W. A. & Jones, D. T. The PSIPRED Protein Analysis Workbench: 20 years on. *Nucleic Acids Res* **47**, W402–W407 (2019).
14. Käll, L., Krogh, A. & Sonnhammer, E. L. L. Advantages of combined transmembrane topology and signal peptide prediction--the Phobius web server. *Nucleic Acids Res* **35**, W429-432 (2007).
15. Almagro Armenteros, J. J. *et al.* SignalP 5.0 improves signal peptide predictions using deep neural networks. *Nat Biotechnol* **37**, 420–423 (2019).
16. Du, Z. *et al.* The trRosetta server for fast and accurate protein structure prediction. *Nat Protoc* **16**, 5634–5651 (2021).
17. Shen, T. *et al.* When homologous sequences meet structural decoys: Accurate contact prediction by tFold in CASP14-(tFold for CASP14 contact prediction). *Proteins* **89**, 1901–1910 (2021).
18. Bittrich, S. *et al.* RCSB Protein Data Bank: Improved Annotation, Search, and Visualization of Membrane Protein Structures Archived in the PDB. *Bioinformatics* btab813 (2021) doi:10.1093/bioinformatics/btab813.
19. Holm, L. DALI and the persistence of protein shape. *Protein Sci* **29**, 128–140 (2020).
20. Ben Chorin, A. *et al.* ConSurf-DB: An accessible repository for the evolutionary conservation patterns of the majority of PDB proteins. *Protein Sci* **29**, 258–267 (2020).
21. Mirdita, M. *et al. ColabFold - Making protein folding accessible to all.* http://biorxiv.org/lookup/doi/10.1101/2021.08.15.456425 (2021) doi:10.1101/2021.08.15.456425.

22. Jumper, J. *et al.* Highly accurate protein structure prediction with AlphaFold. *Nature* **596**, 583–589 (2021).
23. Baek, M. *et al.* Accurate prediction of protein structures and interactions using a three-track neural network. *Science* **373**, 871–876 (2021).
24. Perrakis, A. & Sixma, T. K. AI revolutions in biology: The joys and perils of AlphaFold. *EMBO Rep* **22**, e54046 (2021).
