# Supplementary material for: TMEM161B regulates cerebral cortical gyration, Sonic Hedgehog signaling, and ciliary structure in the developing central nervous system
Source: Proc Natl Acad Sci U S A. 2023 Jan 20;120(4):e2209964120. doi: 10.1073/pnas.2209964120 (PMC9942790; doi:10.1073/pnas.2209964120)
Supplement: Supplementary file 2 — Dataset S01 (DOCX) [file pnas.2209964120.sd01.docx]

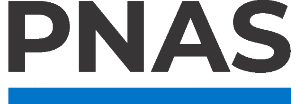


**Undiagnosed Disease Network (UDN) Authors**

The following authors were part of the Undiagnosed Disease Network:

| **First** | **Middle** | **Last** | **Official Institutional Affiliation** |
| --- | --- | --- | --- |
| David | R. | Adams | National Institutes of Health |
| Aaron |  | Aday | Brigham and Women's Hospital |
| Mercedes | E. | Alejandro | Baylor College of Medicine |
| Patrick |  | Allard | University of California Los Angeles |
| Euan | A. | Ashley | Stanford University |
| Mahshid | S. | Azamian | Baylor College of Medicine |
| Carlos | A. | Bacino | Baylor College of Medicine |
| Eva |  | Baker | National Institutes of Health |
| Ashok |  | Balasubramanyam | Baylor College of Medicine |
| Hayk |  | Barseghyan | University of California Los Angeles |
| Gabriel | F. | Batzli | National Institutes of Health |
| Alan | H. | Beggs | Boston Children's Hospital |
| Babak |  | Behnam | National Institutes of Health |
| Hugo | J. | Bellen | Baylor College of Medicine |
| Jonathan | A. | Bernstein | Stanford University |
| Gerard | T. | Berry | Boston Children's Hospital |
| Anna |  | Bican | Vanderbilt University Medical Center |
| David | P. | Bick | HudsonAlpha Institute for Biotechnology |
| Camille | L. | Birch | HudsonAlpha Institute for Biotechnology |
| Devon |  | Bonner | Stanford University |
| Braden | E. | Boone | HudsonAlpha Institute for Biotechnology |
| Bret | L. | Bostwick | Baylor College of Medicine |
| Lauren | C. | Briere | Massachusetts General Hospital |
| Elly |  | Brokamp | Vanderbilt University Medical Center |
| Donna | M. | Brown | HudsonAlpha Institute for Biotechnology |
| Matthew |  | Brush | Oregon Health and Science University |
| Elizabeth | A. | Burke | National Institutes of Health |
| Lindsay | C. | Burrage | Baylor College of Medicine |
| Manish | J. | Butte | University of California Los Angeles |
| Shan |  | Chen | Baylor College of Medicine |
| Gary | D. | Clark | Baylor College of Medicine |
| Terra | R. | Coakley | Stanford University |
| Laurel | A. | Cobban | Brigham and Women's Hospital |
| Joy | D. | Cogan | Vanderbilt University Medical Center |
| Heather | A. | Colley | National Institutes of Health |
| Cynthia | M. | Cooper | Massachusetts General Hospital |
| Heidi |  | Cope | Duke University Medical Center |
| William | J. | Craigen | Baylor College of Medicine |
| Precilla |  | D'Souza | National Institutes of Health |
| Mariska |  | Davids | National Institutes of Health |
| Jean | M. | Davidson | Stanford University |
| Jyoti | G. | Dayal | National Institutes of Health |
| Esteban | C. | Dell'Angelica | University of California Los Angeles |
| Shweta | U. | Dhar | Baylor College of Medicine |
| Katrina | M. | Dipple | University of California Los Angeles |
| Naghmeh |  | Dorrani | University of California Los Angeles |
| Daniel | C. | Dorset | HudsonAlpha Institute for Biotechnology |
| Jessica |  | Douglas | Boston Children's Hospital |
| Emilie | D. | Douine | University of California Los Angeles |
| David | D. | Draper | National Institutes of Health |
| Annika | M. | Dries | Stanford University |
| David | J. | Eckstein | National Institutes of Health |
| Lisa | T. | Emrick | Baylor College of Medicine |
| Christine | M. | Eng | Baylor College of Medicine |
| Gregory | M. | Enns | Stanford University |
| Ascia |  | Eskin | University of California Los Angeles |
| Cecilia |  | Esteves | Harvard Medical School |
| Tyra |  | Estwick | National Institutes of Health |
| Liliana |  | Fernandez | Stanford University |
| Carlos |  | Ferreira | National Institutes of Health |
| Elizabeth | L. | Fieg | Brigham and Women's Hospital |
| Paul | G. | Fisher | Stanford University |
| Brent | L. | Fogel | University of California Los Angeles |
| Noah | D. | Friedman | Stanford University |
| William | A. | Gahl | National Institutes of Health |
| Rena | A. | Godfrey | National Institutes of Health |
| Alica | M. | Goldman | Baylor College of Medicine |
| David | B. | Goldstein | Columbia University |
| Sarah | E. | Gould | National Institutes of Health |
| Jean-Philippe | F. | Gourdine | Oregon Health and Science University |
| Catherine | A. | Groden | National Institutes of Health |
| Andrea | L. | Gropman | National Institutes of Health |
| Melissa |  | Haendel | Oregon Health and Science University |
| Rizwan |  | Hamid | Vanderbilt University Medical Center |
| Neil | A. | Hanchard | Baylor College of Medicine |
| Frances |  | High | Massachusetts General Hospital |
| Ingrid | A. | Holm | Harvard Medical School |
| Jason |  | Hom | Stanford University |
| Ellen | M. | Howerton | National Institutes of Health |
| Yong |  | Huang | Stanford University |
| Fariha |  | Jamal | Baylor College of Medicine |
| Yong-hui |  | Jiang | Duke University Medical Center |
| Jean | M. | Johnston | National Institutes of Health |
| Angela | L. | Jones | HudsonAlpha Institute for Biotechnology |
| Lefkothea |  | Karaviti | Baylor College of Medicine |
| Emily |  | Kelley | Harvard Medical School |
| David | M. | Koeller | Oregon Health and Science University |
| Isaac | S. | Kohane | Harvard Medical School |
| Jennefer | N. | Kohler | Stanford University |
| Susan |  | Korrick | Brigham and Women's Hospital |
| Mary |  | Kozuira | Vanderbilt University Medical Center |
| Donna | M. | Krasnewich | National Institutes of Health |
| Joel | B. | Krier | Brigham and Women's Hospital |
| Jennifer | E. | Kyle | Pacific Northwest National Laboratory |
| Seema | R. | Lalani | Baylor College of Medicine |
| C. | Christopher | Lau | National Institutes of Health |
| Jozef |  | Lazar | HudsonAlpha Institute for Biotechnology |
| Kimberly |  | LeBlanc | Harvard Medical School |
| Brendan | H. | Lee | Baylor College of Medicine |
| Hane |  | Lee | University of California Los Angeles |
| Shawn | E. | Levy | HudsonAlpha Institute for Biotechnology |
| Richard | A. | Lewis | Baylor College of Medicine |
| Sandra | K. | Loo | University of California Los Angeles |
| Joseph |  | Loscalzo | Brigham and Women's Hospital |
| Richard | L. | Maas | Brigham and Women's Hospital |
| Ellen | F. | Macnamara | National Institutes of Health |
| Calum | A. | MacRae | Brigham and Women's Hospital |
| Valerie | V. | Maduro | National Institutes of Health |
| Marta | M. | Majcherska | Stanford University |
| May Christine | V. | Malicdan | National Institutes of Health |
| Laura | A. | Mamounas | National Institutes of Health |
| Teri | A. | Manolio | National Institutes of Health |
| Thomas | C. | Markello | National Institutes of Health |
| Ronit |  | Marom | Baylor College of Medicine |
| Martin | G. | Martin | University of California Los Angeles |
| Julian | A. | Martínez-Agosto | University of California Los Angeles |
| Shruti |  | Marwaha | Stanford University |
| Thomas |  | May | HudsonAlpha Institute for Biotechnology |
| Allyn |  | McConkie-Rosell | Duke University Medical Center |
| Colleen | E. | McCormack | Stanford University |
| Alexa | T. | McCray | Harvard Medical School |
| Jason | D. | Merker | Stanford University |
| Thomas | O. | Metz | Pacific Northwest National Laboratory |
| Matthew |  | Might | Harvard Medical School |
| Ganesh |  | Mochida | Boston Children's Hospital |
| Paolo | M. | Moretti | Baylor College of Medicine |
| Marie |  | Morimoto | National Institutes of Health |
| John | J. | Mulvihill | National Institutes of Health |
| David | R. | Murdock | Baylor College of Medicine |
| Jennifer | L. | Murphy | National Institutes of Health |
| Donna | M. | Muzny | Baylor College of Medicine |
| Michele | E. | Nehrebecky | National Institutes of Health |
| Stan | F. | Nelson | University of California Los Angeles |
| J. | Scott | Newberry | HudsonAlpha Institute for Biotechnology |
| John | H. | Newman | Vanderbilt University Medical Center |
| Sarah | K. | Nicholas | Baylor College of Medicine |
| Donna |  | Novacic | National Institutes of Health |
| Jordan | S. | Orange | Baylor College of Medicine |
| James | P. | Orengo | Baylor College of Medicine |
| J. | Carl | Pallais | Massachusetts General Hospital |
| Christina | GS. | Palmer | University of California Los Angeles |
| Jeanette | C. | Papp | University of California Los Angeles |
| Neil | H. | Parker | University of California Los Angeles |
| Loren | DM. | Pena | Duke University Medical Center |
| John | A. | Phillips III | Vanderbilt University Medical Center |
| Jennifer | E. | Posey | Baylor College of Medicine |
| John | H. | Postlethwait | University of Oregon |
| Lorraine |  | Potocki | Baylor College of Medicine |
| Barbara | N. | Pusey | National Institutes of Health |
| Genecee |  | Renteria | University of California Los Angeles |
| Chloe | M. | Reuter | Stanford University |
| Lynette |  | Rives | Vanderbilt University Medical Center |
| Amy | K. | Robertson | Vanderbilt University Medical Center |
| Lance | H. | Rodan | Boston Children's Hospital |
| Jill | A. | Rosenfeld | Baylor College of Medicine |
| Jacinda | B. | Sampson | Stanford University |
| Susan | L. | Samson | Baylor College of Medicine |
| Kelly |  | Schoch | Duke University Medical Center |
| Daryl | A. | Scott | Baylor College of Medicine |
| Lisa |  | Shakachite | Vanderbilt University Medical Center |
| Prashant |  | Sharma | National Institutes of Health |
| Vandana |  | Shashi | Duke University Medical Center |
| Rebecca |  | Signer | University of California Los Angeles |
| Edwin | K. | Silverman | Brigham and Women's Hospital |
| Janet | S. | Sinsheimer | University of California Los Angeles |
| Kevin | S. | Smith | Stanford University |
| Rebecca | C. | Spillmann | Duke University Medical Center |
| Joan | M. | Stoler | Boston Children's Hospital |
| Nicholas |  | Stong | Duke University Medical Center |
| Jennifer | A. | Sullivan | Duke University Medical Center |
| David | A. | Sweetser | Massachusetts General Hospital |
| Queenie | K.-G. | Tan | Duke University Medical Center |
| Wen-Haan |  | Tan | Boston Children's Hospital |
| Cynthia | J. | Tifft | National Institutes of Health |
| Camilo |  | Toro | National Institutes of Health |
| Alyssa | A. | Tran | Baylor College of Medicine |
| Tiina | K. | Urv | National Institutes of Health |
| Eric |  | Vilain | University of California Los Angeles |
| Tiphanie | P. | Vogel | Baylor College of Medicine |
| Daryl | M. | Waggott | Stanford University |
| Colleen | E. | Wahl | National Institutes of Health |
| Melissa |  | Walker | Massachusetts General Hospital |
| Nicole | M. | Walley | Duke University Medical Center |
| Jijun |  | Wan | University of California Los Angeles |
| Michael | F. | Wangler | Baylor College of Medicine |
| Patricia | A. | Ward | Baylor College of Medicine |
| Katrina | M. | Waters | Pacific Northwest National Laboratory |
| Bobbie-Jo | M. | Webb-Robertson | Pacific Northwest National Laboratory |
| Monte |  | Westerfield | University of Oregon |
| Matthew | T. | Wheeler | Stanford University |
| Anastasia | L. | Wise | National Institutes of Health |
| Lynne | A. | Wolfe | National Institutes of Health |
| Elizabeth | A. | Worthey | HudsonAlpha Institute for Biotechnology |
| Shinya |  | Yamamoto | Baylor College of Medicine |
| Yaping |  | Yang | Baylor College of Medicine |
| John |  | Yang | National Institutes of Health |
| Amanda | J. | Yoon | University of California Los Angeles |
| Guoyun |  | Yu | National Institutes of Health |
| Diane | B. | Zastrow | Stanford University |
| Chunli |  | Zhao | Stanford University |
| Allison |  | Zheng | University of California Los Angeles |
